# Siibra: a software tool suite for realizing a Multilevel Human Brain Atlas from complex data resources

**DOI:** 10.1038/s41592-026-03159-x

**Published:** 2026-07-20

**Authors:** Timo Dickscheid, Xiaoyun Gui, Ahmet N. Simsek, Christian Schiffer, Jean-Francois Mangin, Yann Leprince, Viktor Jirsa, Jan G. Bjaalie, Trygve B. Leergaard, Sebastian Bludau, Katrin Amunts

**Affiliations:** 1 Institute of Neuroscience and Medicine (INM-1), Research Centre Jülich, Jülich, Germany; 2 Institute of Computational Visualistics, University of Koblenz, Koblenz, Germany; 3 Helmholtz AI, Research Centre Jülich, Jülich, Germany; 4 NeuroSpin, CEA, Université Paris-Saclay, Gif-sur-Yvette, France; 5 Institut de Neurosciences des Systèmes, INSERM, Aix-Marseille University, Marseille, France; 6 Institute of Basic Medical Sciences, University of Oslo, Oslo, Norway; 7 Medical Faculty & University Hospital Düsseldorf - Cécile & Oskar Vogt Institute of Brain Research, Heinrich Heine University, Düsseldorf, Germany

**Keywords:** Computational neuroscience, Data integration, Software, Neuroscience

## Abstract

Computational technology opens new possibilities toward understanding the complexity of the human brain, but it requires integrating measurements from different modalities and scales in an anatomical context and exposing them in an interoperable, actionable form. Especially with growing big data resources, accessing information from different scales and modalities coherently for visual exploration, reproducible analysis and application development remains challenging. Here we present siibra, a tool suite that connects diverse data from cloud resources to reference atlases and coordinate spaces. It supports different use cases by making contents accessible through a web viewer, Python library and HTTP application programming interface. Using siibra we implemented a Multilevel Human Brain Atlas linking macro-anatomical concepts and their inter-subject variability with measurements of the microstructural composition and intrinsic variance of brain regions, building on cytoarchitecture as a reference and supporting MRI-based and microscopic templates. The atlas is integrated with the EBRAINS research infrastructure. All software and content are openly accessible.

## Abstract (web summary)

Siibra is a software suite for working with diverse human brain atlases. It links data acquired with different modalities and at different resolution, thereby creating a Multilevel Human Brain Atlas.

## Main

Decoding the human brain requires consideration of multiple levels of structural and functional organization, using measurements obtained at different spatiotemporal scales with complementary methods. To describe the composition and intrinsic variance of brain regions in conjunction with brain-wide neural network connectivity and inter-subject variability, macro-anatomical concepts need to be linked with microstructural features at a cellular resolution. The integration of such comprehensive information requires reference atlases providing consistent and accurate definitions of anatomical structures across a range of spatial resolutions and modalities in multiple reference coordinate systems, and a consistent model for diverse representations of locations in the brain (for example, brain areas, coordinates, or bounding boxes) that reflect how measurements are linked to anatomy. Such integration of reference atlases with datasets across scales and modalities constitutes a multilevel atlas. Its implementation builds on existing concepts, such as those introduced in ref. ^1^, and imposes new conceptual and technical difficulties. In particular, it must accommodate rapidly evolving computational technologies and automated workflows to ensure efficient collection and processing of the comprehensive information it offers. Although originating from very different data sources, elements of the multilevel atlas must be exposed in well harmonized, interoperable and actionable formats to make them available in a computationally scalable fashion.

Ample data resources exist that describe different aspects of brain organization^2^, and a broad range of reference atlases are available reflecting complementary principles of brain organization, typically provided in the form of MRI-scale image volumes or labeled surface meshes at spatial resolutions in the millimeter range derived from in vivo imaging. Integration of information into neuroscience workflows has so far been mostly addressed by casting high-resolution measurements into data representations for macroscopic analyses^3,4^, such as interpolated and averaged feature distributions in surface and voxel formats. Such derivative data make high-resolution features accessible and computable but may reduce the rich regional variance and spatial specificity encoded in the original measurements. In fact, the neuroscience community is increasingly motivated to share primary data with a full level of detail and enable their reuse^5^. This is supported by the increasing attention of publishers to this topic, leading to a growing abundance of large primary data resources on different online repositories. However, accessing and using data resources from different scales and modalities at their full level of detail remains a technical challenge. An important reason for this is the lack of coherent spatial and semantic integration. The structural relationships between, for example, microscopic measurements from histological studies and whole-brain neuroimaging measurements are often unclear, and the data may refer to different versions and representations of information in reference atlases. In addition, links between measurements and brain areas, such as the label indices in a parcellation map, get easily lost during analysis workflows. Moreover, the consistent interpretation of spatial locations in the brain requires accounting for localization accuracy, different spatial resolutions, and effects of transformations between reference spaces. Another difficulty is the fundamental difference in handling big data, such as whole brain sections at full resolution and three-dimensional (3D) volumes from microscopic studies in the giga- to terabyte range, as compared to data being shared in the form of small files.

Progress in obtaining and analyzing large datasets and the need to link the different modalities across spatial scales imply a paradigm shift from downloading files to interacting with cloud resources and services. Researchers are confronted with new and variable data representations and substantially different tool chains. Therefore, to enhance usability and facilitate data use, we need software solutions that expose data from large cloud resources together with classical file-oriented representations in a unified and interoperable fashion and provide operational principles to connect them with workflows for analysis and modeling.

Here we present siibra (‘software interfaces for interacting with brain atlases’), a tool suite designed to tackle these challenges by offering an informatics framework that integrates data from different modalities and resources with reference atlases spanning macroscopic and microscopic resolutions. The tool suite includes an interactive 3D web viewer (siibra-explorer), an HTTP application programming interface (API; siibra-api) and a Python library (siibra-python). It enables us to specify and process various forms of locations in the brain and assign them to brain areas defined in reference atlases through multilayered semantic and spatial relationships, enabling anatomical characterization and multimodal profiling of regions of interest. Relationships between data, spatial locations and brain areas are modeled as associations with a quantifiable degree of uncertainty and evaluated according to accepted reference coordinate systems and brain region taxonomies. We have developed siibra to implement a Multilevel Human Brain Atlas integrated with the EBRAINS research infrastructure (https://ebrains.eu). It combines a complementary selection of reference atlases and coordinate spaces with a broad range of multimodal datasets, supporting voxel and surface-based workflows for the whole brain while offering access to underlying data at microscopic detail. We here present the tool suite and demonstrate its functionalities using the Multilevel Human Brain Atlas as a challenging use case. By including the underlying executable code or persistent URLs with each figure, the present paper also serves as a practical demonstration of how siibra enables fully reproducible workflows that are easily accessible to all researchers.

## Results

**Fig. 1:**
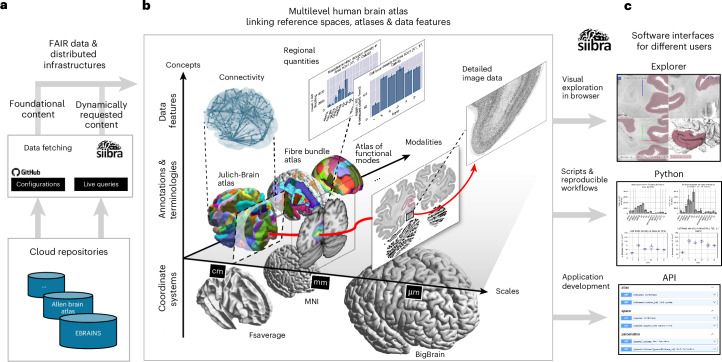
The siibra tool suite implements a Multilevel Human Brain Atlas from complex, multiscale data resources. **a**, Contents of the multilevel atlas are curated and stored in cloud repositories, with EBRAINS (https://ebrains.eu) chosen as the main platform following the openMINDS metadata framework (https://github.com/openMetadataInitiative/openMINDS). We distinguish foundational content, predefined as versioned collections of configuration files, from dynamic content obtained at runtime using ‘live queries’ to APIs of selected cloud repositories. **b**, The tool suite links comprehensive data resources with reference atlases into the Multilevel Human Brain Atlas, covering different reference coordinate systems and atlas annotations which capture inter-subject variability as well as microscopic details of individual brains. Brain areas and spatial locations are linked with multimodal datasets, including microscopic images, connectivity matrices and regional quantities such as neurotransmitter receptor densities. The red arrow illustrates a typical access path from a brain area to a detailed measurement in a relevant dataset. **c**, The tool suite is a modular environment consisting of cloud services and installable software components. It provides access to all contents of the Multilevel Human Brain Atlas in harmonized, actionable data formats through interactive and programmatic user interfaces.

In the following, we demonstrate how key functionalities of siibra enable the implementation of a Multilevel Human Brain Atlas, linking reference atlases and comprehensive datasets from cloud resources and making the underlying data accessible and actionable for neuroscience applications (Fig. 1). All data and functionality are openly accessible. References to corresponding executable codes and online resources are provided throughout the text to ensure reproducibility.

The work relies on several key concepts, whose distinctions are crucial for understanding the presented results and methodology. First, we distinguish individual reference atlases from the overarching concept of the Multilevel Human Brain Atlas. The latter combines complementary reference atlases reflecting different principles of brain organization (Fig. 1b), such as the Julich-Brain atlas (cytoarchitecture^6^), the deep white matter bundle atlas (fiber architecture^7^) or the atlas of dictionaries of functional modes (functional architecture^8^). Furthermore, following the AtOM ontology model^9^, we assume that each individual reference atlas provides a terminology of brain area definitions, often in hierarchical form. Brain areas are delineated in images or surfaces of different reference coordinate systems, resulting in annotation sets. Annotations may be given in the form of labeled or statistical maps, reflecting discrete or continuous annotations respectively. Discrete annotation sets covering the whole brain are often further denoted as parcellation maps.

### Design and architecture of siibra

The siibra tool suite is a modular environment consisting of cloud services and installable software components (Fig. 1). It includes an interactive 3D web viewer (siibra-explorer), an HTTP API (siibra-api), and a Python library (siibra-python). In either form, it assumes minimal technical requirements: The online viewer can be accessed with common web browsers, including mobile devices, and co-display user-supplied NIfTI files with atlas content without uploading them. The Python client can be installed with established package managers from https://pypi.org/project/siibra and provides a broad range of documented code examples at https://siibra-python.readthedocs.io/en/v1/examples.html.

**Extended Data Fig. 1:**
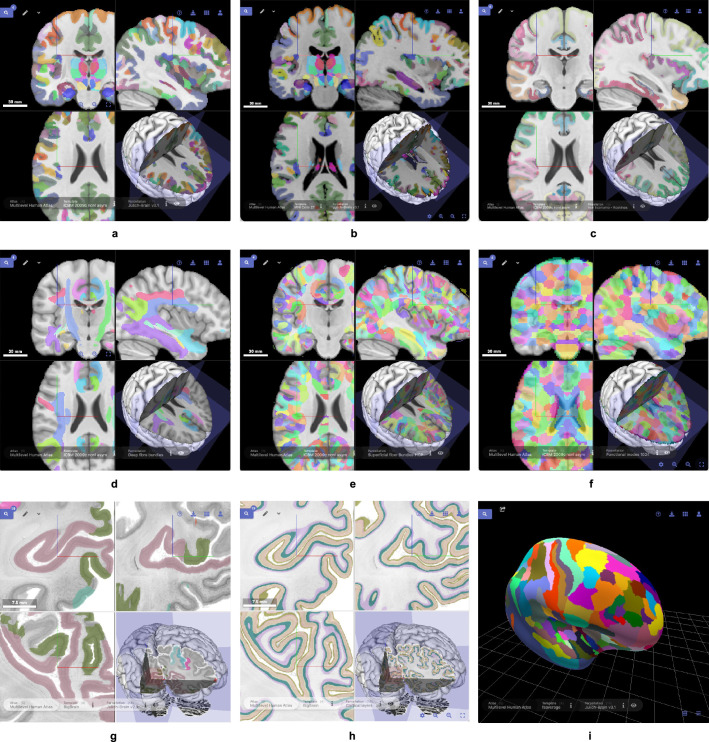
Selected reference parcellation maps of the Multilevel Human Brain Atlas displayed in siibra-explorer. Screenshots of siibra-explorer displaying a selection of parcellation maps from the default configuration of the Multilevel Human Brain Atlas. **a, b**, **g, i**, Variants of Julich-Brain cytoarchitectonic maps in different reference spaces. **c**, von Economo map. **d**, **e**, Deep white matter and superficial bundle maps. **f**, DiFuMo map of 1024 functional modes. **h**, BigBrain cortical layer maps. Maps are shown in ICBM 2009c asymmetric space, except **b** (MNI Colin 27 space), **g,** **h** (BigBrain histological space) and **i** (fsaverage surface space). Extended Data Table 1 provides a full list of currently available maps with references.

**Extended Data Fig. 2:**
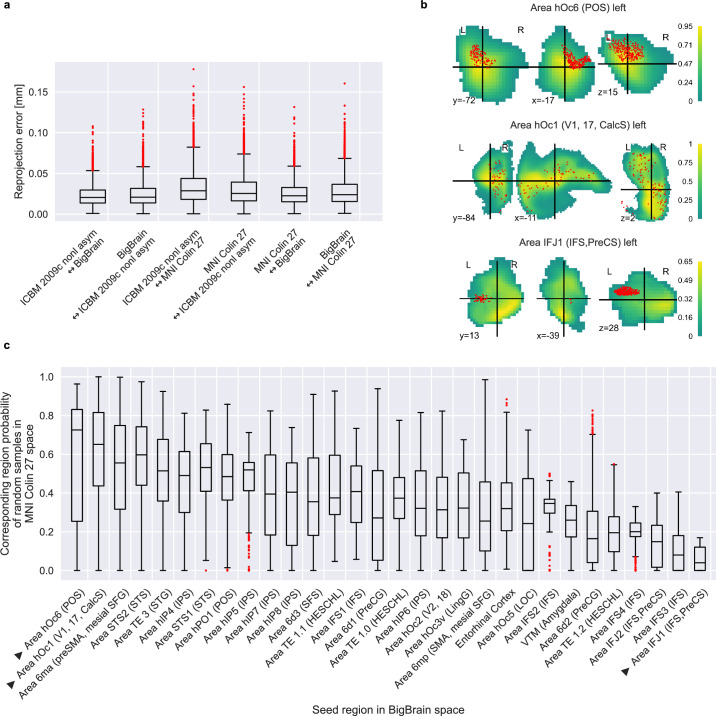
Quantitative assessment of coordinate warping applied in siibra. (**a**) Distribution of reprojection errors (in mm) after warping n=5000 randomly sampled gray matter locations from a source to a target reference space (box plots; center line: median; box limits: 25th/75th percentiles; whiskers: 1.5x interquartile range; points beyond whiskers: outliers). While the underlying nonlinear transformations are diffeomorphisms, numerical inaccuracies in the order 10-20 micrometer have to be expected, occasionally reaching up to about 100 micrometer. (**b**, **c**) Warping of n=500 random coordinates sampled uniformly from each ultrahigh-resolution cytoarchitectonic map in BigBrain space to MNI ICBM 2009c asymmetric space. (**b**) Plot of warped random samples for three example regions on top the respective probability maps to illustrate the variation (region selection marked by black triangle in c). (**c**) Probabilities obtained by re-assigning the warped coordinates to the same brain region using Julich-Brain probabilistic maps in MNI space (box plots; centre line: median; box limits: 25th/75th percentiles; whiskers: 1.5x interquartile range; points beyond whiskers: outliers).

The tool suite does not store any actual data itself. Instead, all atlas elements and data features (referred to as siibra content) are tagged with comprehensive metadata and point to published data resources (Fig. 1a). This includes reference atlases and coordinate systems, as specified in Extended Data Table 1 and Extended Data Fig. 1 for the Multilevel Human Brain Atlas, as well as a selection of multimodal datasets linked to these. Because content is separated from software, installation of the Python client requires only minimal storage space. Following a lazy data-fetching strategy, siibra downloads only requested data elements to gradually build up a configurable local data cache according to user needs. For operating the system in commercial or clinical environments with restricted network policies, the complete tool suite can be set up for offline use by cloning the configuration to a local computer, pre-populating the cache explicitly and deploying the API and viewer components as local network services.

siibra enables specifying and processing various forms of spatial locations in the brain, such as centers of gravity of measured neuroimaging signals, coordinates of functional activations and bounding boxes of regions of interest in brain tissue. These locations can be transformed between reference coordinate systems using precomputed diffeomorphic transformations, with an expected accuracy depending on the degree of nonlinear deformation in the respective region (Extended Data Fig. 2).

**Extended Data Fig. 3:**
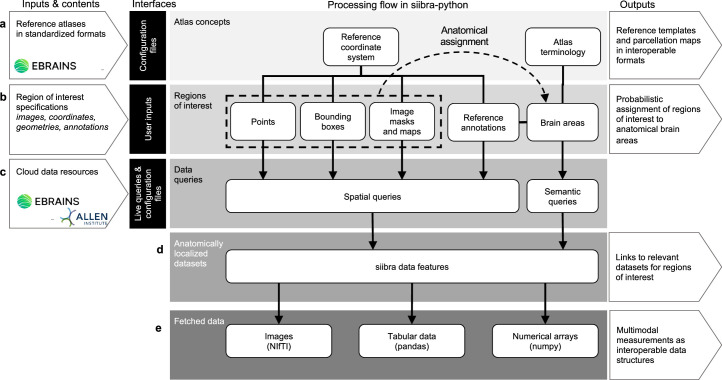
High-level illustration of siibra-python’s system architecture. The siibra-python library models elements of reference atlases and spatial locations to enable assignment of regions of interest to brain areas, and implements queries for retrieving anatomically localized data features from cloud resources. (**a**) Atlas elements include reference coordinate spaces and parcellations with corresponding terminologies, which are stored in standardized form in data repositories such as EBRAINS and linked to siibra through configuration files. The library provides efficient and reproducible retrieval of maps and templates at different spatial resolutions in interoperable formats such as NIfTI. (**b**) Users can specify regions of interest by providing images, coordinates, geometric shapes or textual annotations, which siibra assigns to brain areas via probabilistic reference maps. Resulting assignments can be accessed and reused as pandas DataFrames. (**c**) Regions of interest and brain areas can then be used to run queries to cloud repositories to obtain customized multimodal profilings. Foundational datasets are linked to siibra in the form of configuration files, and complemented with dynamic content resulting from API calls (‘live queries’), currently including interfaces to EBRAINS and the Allen microarray data for gene expressions in the human brain. (**d**) Data queries result in data features, which can be considered as enriched links between anatomical locations in siibra and files published on the respective online resource. The links can be accessed in the form of URLs and DOIs together with a qualification of their anatomical anchoring to the atlas. **e**: By fetching from a data feature, siibra provides access to the underlying data in interoperable formats such as NIfTI images, pandas DataFrames, or numpy arrays for simple reuse. Following a lazy loading scheme, data is retrieved from the cloud resource only when required, and then cached for future requests.

Locations can be assigned to brain areas defined in the reference atlases through multilayered semantic and spatial relationships, and used to retrieve multimodal data features connected to these structures. Content elements are then fetched and exposed by siibra in interoperable formats (Extended Data Fig. 3).

**Extended Data Fig. 4:**
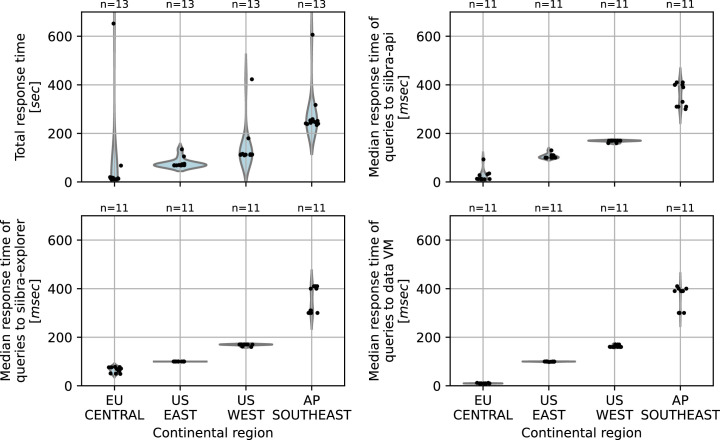
Response times of a typical access pattern from different continents. Distribution of measured response times when performing the scenario in Fig. 2 in siibra repeatedly from different continents. Data are displayed as Kernel density estimates of the full distribution with individual data points (number listed as ‘n’) shown as jittered dots. Measurements were carried out by recording the HTTP requests of the workflow as an Ansible playbook, and running the same requests from virtual ma- chines (1 CPU, 1GB RAM, 40 Gbps / 1 Gbps network in/out) provisioned on Linode located on pre-specified geographical locations. Playbooks were executed at least ten times on different dates during August and September 2025. We report the total response time in milliseconds of each playbook run. From top left to bottom right: Total response times, response times to the subset of requests to siibra-api, siibra-explorer and underlying virtual machines serving data.

Due to the distributed nature of the underlying data resources, response times for data retrieval can vary depending on the location of the user and the server hosting the data. At present, most of siibra’s contents are hosted on EBRAINS, which uses storage systems on European supercomputing centers. We analyzed response times when running a typical workflow repeatedly from different geolocations, and found that the performance of siibra is usually fluid and reliable inside Europe, but could be unsatisfactory from other continents including North America, Asia and Australia (Extended Data Fig. 4).

Further details of the software architecture design are described in the Methods section.

### The Multilevel Human Brain Atlas

We have developed siibra to implement a multilevel atlas of the human brain that is integrated with the EBRAINS research infrastructure (https://ebrains.eu) and provides interfaces to additional data repositories. This atlas combines reference atlases and coordinate spaces at different scales (Extended Data Table 1 and Extended Data Fig. 1) with multimodal datasets (Extended Data Table 2), supporting voxel and surface-based workflows for the whole brain while offering access to underlying data at microscopic detail.

The reference spaces include at the macroscopic scale the International Consortium for Brain Mapping (ICBM) 2009c nonlinear asymmetric multisubject and the Colin27 single-subject average templates^10^ as well as surface subspaces (fsaverage and fsaverage6) from the Freesurfer software suite^11,12^. These are combined with the BigBrain space at microstructural level^13^, which represents a 3D reconstruction of 7,404 histological sections from an individual postmortem human brain with an isotropic voxel resolution of 20 × 20 × 20 μm.

A central element of the Multilevel Human Brain Atlas is the Julich-Brain cytoarchitectonic atlas^6^, which includes probabilistic maps of more than two hundred cortical and subcortical areas for each hemisphere to date. The Julich-Brain atlas was chosen due to its rigorous multiscale approach: (1) it captures regional microstructural variance based on studies of ten postmortem brains; (2) it provides reproducible, observer-independent mapping; (3) the quality of the mapping has been validated through numerous peer-reviewed publications for all areas; and (4) it includes a microscopic template space, the BigBrain, enabling to bridge the macro scale (as represented in the MNI template) with the micro scale (as represented in BigBrain). An increasing number of mapped areas are annotated as detailed 3D maps across the entire series of BigBrain histological sections with 20-μm isotropic resolution^14^, supplemented by a deep learning workflow. Deep learning was instrumental in speeding up mapping in intermediate sections to achieve dense segmentation of areas over their full extent. In line with our vision of a ‘living atlas’, updates with new areas are being released at a rate of 1–2 per year. Such thorough mapping remains very time-consuming, with an estimated effort of one area per year and scientist.

The cerebral cortex in the BigBrain is further segmented by maps of six isocortical layers based on an automatic classification of 3D cortical intensity profiles^15^. The cytoarchitectonic maps are aligned with maps of superficial as well as long white matter bundles^7,16^ obtained by clustering streamlines from diffusion-weighted imaging of a large number of subjects, as well as maps of functional modules at different granularities computed from millions of fMRI scans to provide an efficient reduction and representation of BOLD signals^8^.

The data resources currently connected to the multilevel atlas include detailed measures of cellular (for example cell densities and staining profiles) and molecular (for example receptor densities and gene expressions) architecture, parcellation-based structural and functional connectivity from neuroimaging cohorts, and an extensive collection of high-resolution histological measurements (Extended Data Table 2).

### Visually guided exploration from full brain networks to cells

The interactive atlas viewer siibra-explorer allows visually guided exploration of brain organization from the macroscopic scale of the brain down to individual cell bodies, and can be accessed with any web browser, including mobile touchscreen devices. It integrates very large and detailed image resources (Extended Data Table 2) into a 3D visualization of the whole brain. In addition, it embeds cross-sectional volumetric and surface views into an interactive workflow that allows us to navigate reference atlases in different coordinate systems, and to access multimodal datasets anchored to brain areas or coordinate-based locations. Cross-sectional views can be adjusted in real-time to display arbitrary oblique cutting planes and zoom levels, which is crucial, for example, for studying the cortical laminar structure of a highly folded cortical surface. Individual planar views can be maximized to obtain a large field of view, and support layering to co-display brain region maps with underlying image data.

**Fig. 2:**
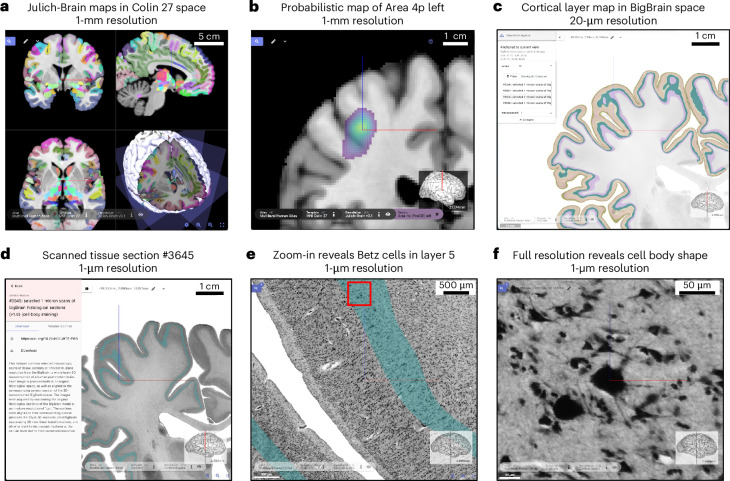
Visually guided exploration of brain structure from the whole brain down to individual cells with siibra-explorer. **a**,**b**, Selection of area 4p in the left hemisphere from the Julich-Brain cytoarchitectonic maps^6^ in MNI Colin 27 space^10^ brings up its probability map. **c**, When switching to the microscopic BigBrain model as a reference space^13^, the level of detail is substantially increased while siibra-explorer preserves zoom and location. The parcellation is changed to cortical layer maps^15^, and anchored image data are revealed in the side panel. **d**, A 1-μm scan of a coronal whole brain section^28^ is selected and superimposed with the 3D model. **e**,**f**, Zooming to full resolution reveals presence and shape of individual cell bodies of Betz giant cells, clearly located in layer V. All steps can be reproduced using the following URLs: **a**, atlases.ebrains.eu/viewer/go/siibra_paper_2A; **b**, atlases.ebrains.eu/viewer/go/siibra_paper_2B; **c**, atlases.ebrains.eu/viewer/go/siibra_paper_2C: **d**, atlases.ebrains.eu/viewer/go/siibra_paper_2D; **e**, atlases.ebrains.eu/viewer/go/siibra_paper_2E; **f**, atlases.ebrains.eu/viewer/go/siibra_paper_2F.

The first example concerns Betz cells in layer V of the primary motor cortex (area 4p^6^). The workflow starts by selecting the MNI Colin27 template and Julich-Brain as reference atlas, displayed as a cross-planar view combined with a rotatable 3D surface visualization in the corresponding reference coordinate system (Fig. 2a). By maximizing one of the cutting planes, a more focused view is obtained and can be adjusted to display an oblique sectioning angle (Fig. 2b).

When switching to the BigBrain template, precomputed nonlinear spatial transformations are used to preserve location and zoom level while changing the reference coordinate system (Fig. 2c). Besides inevitable trade-offs required in nonlinear cross-subject registration, as described for example in ref. ^17^, the precision of this coordinate translation depends on numerical limitations and the degree of nonlinear deformation between the templates in the zoomed-in region of interest (Extended Data Fig. 2).

If a particular brain area is selected in the source reference space (for example Fig. 2b), siibra-explorer tries to preserve the selection in the target space. However, a corresponding map of the region might not be available in the target space. In this case, the area selection is discarded with a corresponding notification, and the corresponding region of interest is displayed as uncharted in the target reference space.

This mechanism can be used to identify macroscopic structures in MNI space as a starting point for exploring detailed maps of cytoarchitectonic regions and cortical layers in the BigBrain^15^, and finding relevant microscopic image data at cellular resolution anchored to this coordinate space (Fig. 2d). The viewer allows us to select microscopic images and superimpose them with the underlying template, so that they can be explored at full spatial resolution while keeping linked with the whole brain context (Fig. 2e,f). The multilayered views can be reused by bookmarking the browser URL or downloading the underlying zoomed-in data, using the download button on the top right.

### Anatomical characterization and multimodal profiling for regions of interest

**Fig. 3:**
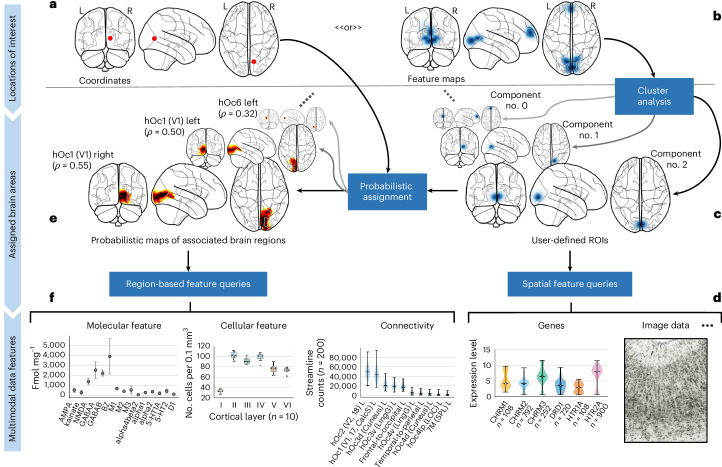
Anatomical characterization and multimodal profiling of regions of interest. **a**,**b**, siibra accepts user-defined location specifications in the form of reference space coordinates with optional certainty quantifiers (**a**) or feature maps from imaging experiments (**b**). **c**, Feature maps are typically separated into cluster components. **d**, Any region of interest (ROI) in image form can be used to run spatial feature queries and extract colocalized multimodal data features. **e**, In addition, locations of interest are assigned to brain areas using probabilistic maps from functional, cyto- or fiber architectonic reference atlases, distinguishing incidence, correlation and overlap of structures. **f**, Resulting associated brain areas reveal additional relevant data features. Fig. 4 provides details regarding the statistical plots in **d**,**f**. The figure can be reproduced using the tutorial notebook available in the online documentation^56^.

The siibra tool suite enables the assignment of user-defined regions of interest to brain areas from reference atlases and facilitates the retrieval of relevant multimodal measurements. This can be used to further characterize these regions in terms of structure, function and connectivity. In the simplest case, locations are specified as 3D coordinates in a supported reference space (Fig. 3a) using siibra’s interactive or programmatic location definition options. Associated brain regions are then assigned to brain areas from a reference atlas (Fig. 3e) utilizing the available 3D reference maps (for example functional, cyto- or fiber architectonic maps) and distinguishing between incidence, correlation and overlap of structures. In the case of statistical maps, where each brain area is typically represented by a separate image volume to account for the overlap in the maps, this might imply to retrieve, load and process several hundred image volumes. To reduce resulting download times and memory requirements, siibra uses a sparse data representation for statistical maps which minimizes the memory footprint. Associated brain areas can then be used to run data queries and find multimodal data features connected to these structures (Fig. 3f). During this process, siibra automatically resolves spatial relationships between different reference spaces and, if necessary, warps coordinates using precomputed diffeomorphic transformations.

The current set of data features in the Multilevel Human Brain Atlas are grouped into the categories cellular, molecular, fibers, functional, connectivity and macrostructure (Extended Data Table 2). Their content includes combinations of image, tabular and numerical array data. Tabular data structures include cortical density measures from histological labeling, ‘fingerprints’ encoding means and s.d. of densities from tissue samples, structural and functional connectivity matrices, or external data such as gene expression levels from the Allen Human Brain microarray data^18,19^. Connectivity matrices are parcellation-grouped numbers and lengths of streamlines obtained from tractography of diffusion MRI data as well as correlations of resting-state and task-specific functional time series extracted from different neuroimaging cohorts. Image features include tissue sections, volumes of interest from high-resolution imaging experiments, and in vivo neuroimaging scans. Histological image data are typically anchored to the BigBrain template, as it provides the necessary level of detail to embed microscopy data.

In addition to coordinates, a region of interest may be specified by an image volume (Fig. 3b), for example, an activation map from a functional neuroimaging study. By processing segments or certain intensity ranges, images can be directly used for spatial feature queries (Fig. 3c) to access spatially anchored datasets such as co-registered two-dimensional (2D) and 3D image data, or regional quantities in tabular form such as local gene expressions retrieved from the Allen Human Brain Atlas^18,19^. For anatomical assignment, siibra computes correlation and overlap measures between the input image and brain area annotations in a reference atlas. In many cases it is advisable to split such feature maps into cluster components before computing the assignment (Fig. 3c). The given example uses hierarchical density-based clustering^20^ of points sampled from the input image, based on an implementation in scikit-learn (https://scikit-learn.org/stable/; RRID:SCR_002577), which is available through the PointSet class in siibra-python. Note that siibra also uses image-based assignment to brain regions for processing coordinates with high localization uncertainty. This is realized automatically by converting uncertain coordinates into a 3D image of a Gaussian blob with corresponding kernel size.

As a result, for a given region of interest and a selected parcellation terminology, siibra implements two assessments. The first is an anatomical assignment of the region of interest, represented by a list of associated brain areas qualified by means of probabilities, spatial overlap or correlation, and their possible probabilistic maps in the corresponding coordinate space. The second is a multimodal profile, represented by a compilation of data features that characterize the region of interest. To support the FAIR (findable, accessible, interoperable and reusable) principles of data organization, the underlying data elements are exposed in interoperable community formats and explicitly linked to the resource from which the information is retrieved. For example, tabular data are exposed as pandas (https://pandas.pydata.org) DataFrames for optimal compatibility with common statistics and spreadsheet tools, while 3D images are exposed as NIfTI image objects (https://nipy.org/nibabel/nifti_images.html), which are supported by almost any neuroscience application.

In addition to the shown programmatic solution, siibra-explorer offers an interactive way to carry out a similar workflow using only a web browser. This is possible by drag-and-drop of an image volume from the user’s local computer onto the browser window (documented as ‘superimposing local files’ at https://siibra-explorer.readthedocs.io/). Assuming the volume is provided in NIfTI format and prealigned to the selected reference coordinate system, siibra-explorer will display it as an adjustable semitransparent overlay on top of the selected reference atlas, allowing direct visual assessment of overlap between brain areas and peaks or clusters depicted in the input image. Relevant data features can then be selected and downloaded from the side panel of siibra-explorer. The principle is visualized in a screencast video^21^ delete included in the documentation of siibra-explorer; however, the reproducibility of this viewer-based solution depends on the local environment, and is not supported per se in siibra.

### Multimodal comparison of brain areas

**Fig. 4:**
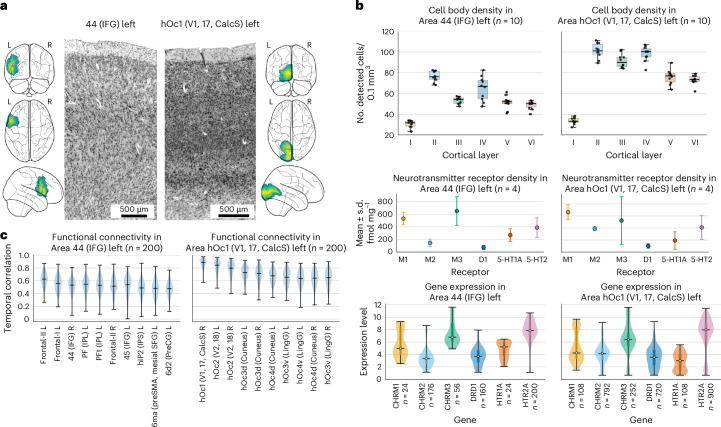
Multimodal comparison of two cortical brain areas. Maps and multimodal regional measurements obtained using siibra-python for language area IFG 44 and primary visual region hOc1d in the cytoarchitectonic reference atlas^6^. **a**, Probabilistic maps in MNI asymmetric space with representative cortical image patches at 1*-*μm resolution extracted from scans of BigBrain sections (image contrast increased^28^) using the principle illustrated in Fig. 5. **b**, Layer-specific densities of cell bodies^57,58^ measured in *n* = 10 different image patches per region obtained from original tissue sections of BigBrain (center line, median; box limits, 25th/75th percentiles; whiskers, 1.5× interquartile range; points beyond whiskers, outliers; individual data points shown as jittered dots) (top). Mean densities and s.d. of a selection of monogenetic neurotransmitter receptors across *n* = 4 tissue samples from three different individuals^59^ (individual data points not published) (middle). Expression levels of a selection of genes coding for these receptors^18,19^ (bottom). The number of data points *n* specified in each column label denotes the number of microarray probe measurements across tissue samples and donors from the Allen Human Brain microarray dataset^18,19^; values range from *n* = 24 to *n* = 900. **c**, Functional connectivity profiles referring to temporal correlation of fMRI time series of 100 individuals from the Human Connectome Project^24,60^ showing the ten strongest connections per brain area for the average connectivity patterns. Violin plots in **b** and **c**, kernel density estimate of the full distribution (center white dot shows median; thick vertical bar shows 25th/75th percentile range; thin vertical line shows min–max range). The figure can be reproduced using the notebook available at ref. ^22^ delete.

A common use case for the aforementioned anatomical assignment and multimodal profiling is to compare architectural features in different regions of interest. We demonstrate the principle by characterizing two cortical areas from the Julich-Brain atlas in terms of cell and neurotransmitter densities, gene expressions and functional connectivity. As an example, data features of area 44 of Broca’s region (IFG) were compared to those of the primary visual cortex (hOc1/BA17/V1), both in the left hemisphere. The same principle can be applied to other brain areas and reference atlases, as well as to user-defined regions of interest. Retrieval of all information is implemented using siibra-python in a Python notebook included in the documentation^22^. The feature plots (Fig. 4) allow for several interesting observations.

**Fig. 5:**
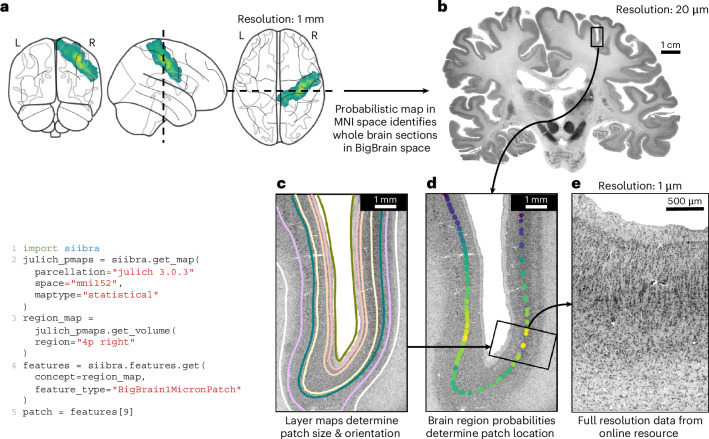
Anatomically guided reproducible extraction of full-resolution image data from cloud resources. **a**,**b**, The code on the left retrieves the probabilistic map of area 4p in the right motor cortex from the cytoarchitectonic atlas^6^ (**a**; ll. 2–3) to sample relevant cortical patches from high-resolution scans of whole-brain tissue sections in BigBrain space^13,28^ (**b**; l. 4). **c**, To define an oriented cortical image patch, siibra automatically loads and intersects cortical layer surface meshes^15^ with each relevant section image. **d**, It then finds points on the intersected layer IV surface with high relevance according to the probability map of the motor area. The different coordinate systems are automatically handled using nonlinear transformations. For a given point on the layer IV surface, siibra finds the closest 3D cortical profile in the layer maps to obtain information about orientation and thickness of the cortex at the chosen position. The 3D profile is projected to the respective image plane and used to define a rectangular patch. **e**, The full-resolution image data in the cortical patch are then fetched from the underlying cloud resource (l. 5). The patch can be used to extract cell body instances and compute quantitative density measures using state-of-the-art instance segmentation models, resulting in cell densities as published in a recent data collection (Fig.span c lass="nonbreakspace" contenteditable="false"> 4b; for example ref. ^57^). The figure can be reproduced using the notebook available in ref. ^61^.

At the cellular level, the two regions show distinct cortical architecture, revealed by different cell body distributions in cortical patches extracted from 1-μm resolution scans of the BigBrain (Figs. 4a and 5). This is reflected quantitatively in the average cell body densities across cortical layers, which differ between the areas, with overall higher values in the visual cortex, in particular the granular layers II and IV (Fig. 4b).

At the molecular level, receptor densities for selected monogenetic receptors (M1, M2, M3, D1, 5-HT1A and 5-HT2) show many similarities between area 44 and area hOc1, but also some notable differences such as a notably higher density of the M2 receptor in hOc1 (Fig. 4b). These receptors represent a subset of up to 19 available receptors, here reduced to those encoded by a single gene. The expression patterns of genes coding for the selected monogenetic receptors (Fig. 4b) match well to the respective receptor densities (Fig. 4b), which is in line with earlier studies highlighting such covariation between the modalities^23^. Gene expressions were retrieved by siibra from the Allen Human Brain Atlas API^18,19^.

Finally, functional connectivity profiles extracted from the Human Connectome Project data^24^ show distinct characteristic relationships for the two regions. For V1, we see strong connectivity into the hierarchy of higher order visual areas, following the organization of the dorsal and ventral streams. For area 44 left, we observe strong temporal correlation with 44 right and 45, which together build the Broca region, as well as to the frontal pole, in correspondence with the known anatomy of the arcuate fasciculus^25^.

Here, we focus on summarizing observations to demonstrate key software functionalities, and refrain from delving into a deeper neuroscientific discussion, which would require additional analyses and statistical tests.

### Anatomically guided reproducible extraction of microscopy data

Besides retrieving of precomputed data features, siibra allows dynamic extraction of high-resolution image data from cloud image resources. For example, image intensities in the BigBrain model^13^ can be interpreted as optical cell densities and thus provide a resource for studying basic aspects of cytoarchitecture. At an isotropic voxel resolution of 20 × 20 × 20 μm, the BigBrain constitutes almost 1 TB of image data, making it infeasible for many users to download and process the complete dataset. Using siibra-explorer, BigBrain can be viewed at full resolution by zooming in with the mouse wheel, such as the example region of interest bookmarked at https://atlases.ebrains.eu/viewer/go/bigbrain_precs_example. By hiding the parcellation map (by clicking the ‘eye’ icon near the parcellation selector, or the ‘y’ key), the image data can be observed at full resolution (Fig. 2c).

However, interactive viewing of image data alone is not the preferred approach for performing reproducible studies and does not scale to large experiments that leverage the potential of big data resources. For this purpose, siibra-python can identify and fetch image data from cloud resources in an efficient and reproducible way. The underlying strategy is to specify a volume of interest using a bounding box or selecting a brain area from a reference atlas, then use siibra-python to identify relevant image datasets and extract the image parts intersecting the volume of interest. This process is facilitated by automatic handling of different coordinate spaces for the query, allowing fetching of data from multiple coordinate spaces using customized resolutions and fields of view, with automatic computation of the spatial metadata arising from this extraction process. As a result, a representative cortical patch at 1-μm resolution can be automatically identified and fetched by selecting a cytoarchitectonic brain area (Fig. 5). The image data are exposed as a NIfTI image annotated with the appropriate transformation matrix into the BigBrain reference coordinate system. As such, the fetched data are suitable for downstream analysis tasks such as cell segmentation, and for combination with other image data referring to supported reference coordinate systems.

### Anatomical evaluation of subcortical maps

To demonstrate the functionalities of siibra, maps of subcortical structures derived from in vivo imaging were reanalyzed to localize them precisely and link them to microstructural structures. The subcortical maps were taken from a recent study^26^, which proposed an in vivo sub-parcellation of the ventral intermediate nucleus (VIM) based on streamlines extracted from diffusion MRI data in humans. In short, the authors of ref. ^26^ extracted the set of streamlines connecting the thalamus with predefined cortical target regions, resulting in structural connectivity profiles for each voxel in the thalamus per subject. Using *k*-means clustering, voxels in the thalamus were then grouped into sub-regions with similar connectivity profiles, resulting in multiple candidate subthalamic maps, here denoted as cluster maps. The study identified cluster maps with high spatial overlap to the VIM, using Dice scores with histology-based segmentation masks of VIM^27^ projected to the individual subject spaces, resulting in a selection of only two relevant cluster maps, Precentral and Dentate.

**Fig. 6:**
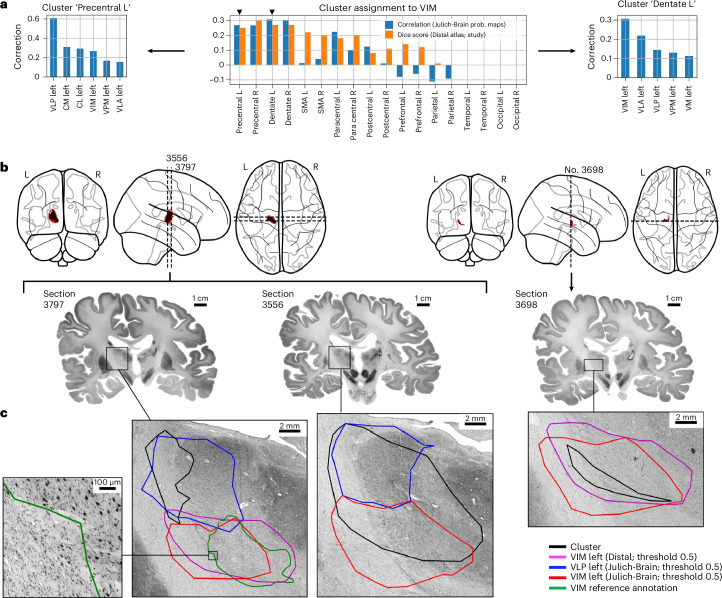
Anatomical evaluation of subcortical maps using siibra-python. **a**, Spatial correspondence of connectivity-based subthalamic cluster maps^26^ with cytoarchitectonically defined nuclei. Dice scores in individual subject space between cluster maps and the VIM in the Distal atlas^27^ reported in ref. ^26^, compared to correlation coefficients with the Julich-Brain probabilistic map of VIM^62^ computed in MNI152 asymmetric space by siibra-python as in Fig. 3 (center). Black triangles indicate the clusters selected for further analysis. Correlation coefficients of clusters Precentral and Dentate (left hemisphere) with other probabilistic maps in Julich-Brain, thresholded at *Ρ* = 0.25 (left and right). **b**, Histological sections from BigBrain^13,28^ identified and retrieved using siibra-python based on the Precentral and Dentate cluster maps as in Fig. 5. **c**, Assessment of the underlying cytoarchitecture and mapped structures at cellular resolution by intersection of maps with the histological sections. In section 3797, the Precentral cluster (black) lies within the VPL (blue) in Julich-Brain while being only adjacent to the VIM (left). Contours of the VIM in Julich-Brain (red) and Distal atlas (magenta) are overall consistent. A reference annotation by a trained neuroanatomist (green) confirms the approximate delineation of VPL and VIM projected from MNI to BigBrain space. Section 3556, located further posterior, shows a less distinct yet confirmative association where both VPL and VIM overlap with the Precentral cluster (center). In section 3698, the Dentate cluster (black) lies clearly inside the borders of the VIM in both Julich-Brain (red) and Distal (magenta) as suggested by the overlap scores in **a** (right). **d**, Full-resolution view of the retrieved image near the VIM boundary in section 3797, revealing clear cytoarchitectonic differences at the reference delineation of the expert (green). The figure can be reproduced using the notebook available in ref. ^63^.

We used siibra-python to reassess the selection using probabilistic assignment (Fig. 3) and to verify the situation at cellular resolution using microscopy data extraction (Fig. 5). The results reveal several interesting aspects (Fig. 6).

Assignment of cluster maps to Julich-Brain probabilistic maps in MNI space independently confirmed the selection of the Precentral and Dentate clusters in the study in terms of high correlation coefficients (Fig. 6a).

For the Dentate cluster, association with VIM was further confirmed as the strongest overall correlation among cytoarchitectonic areas in Julich-Brain (Fig. 6a); however, for the Precentral cluster, the strongest correlation was found with the ventral posterior lateral nucleus (VPL) rather than the VIM (Fig. 6a). This indicates that the Precentral cluster may not be connected to a subpart of VIM, but rather to the adjacent VPL.

The intersection of the different MNI maps with relevant histological sections from the BigBrain model^13,28^ allowed to further examine the localization at cellular resolution (Fig. 6b,c), revealing that the Precentral cluster was indeed located within the borders of VPL, while being only adjacent to VIM (Fig. 6c). Some sections showed a less distinct yet confirmative association where both VPL and VIM overlap with the Precentral cluster (Fig. 6c). In contrast, the Dentate cluster was usually located within the borders of VIM in both Julich-Brain and Distal atlas (Fig. 6c).

The level of detail in the images retrieved with siibra-python allowed to clearly observe the cytoarchitectonic differences defining the boundary of VIM (Fig. 6c, left), enabling a neuroanatomist to create a reference annotation in one of the sections (Fig. 6c). The reference annotation confirmed overall plausible location of the VPL and VIM contours projected from MNI to BigBrain space.

Thus, siibra-python enabled simple and reproducible assignment of the connectivity-based subthalamic maps to cytoarchitectonically defined subcortical structures, confirming some findings of the published study while providing additional, more fine-grained localization details and links to openly accessible reference parcellations. It further provided detailed evidence of the assessment at cellular resolution by facilitating access to relevant histological image data and automatically handling the different coordinate systems and data types involved in the process.

### Expansion with new contents

By maintaining a strict separation from the software, the siibra tool suite allows the content of the Multilevel Human Brain Atlas to continue to expand. In particular, any terminology, parcellation map, reference template or data feature exposed by siibra points to an external resource which contains the actual data (Fig. 1a). This can be a file on a public data repository such as EBRAINS, a segment of a cloud image volume, a local file provided by the user, or data that is dynamically retrieved from a cloud service. We distinguish foundational content, defined as json specifications in a configuration, from dynamically retrieved content obtained through siibra’s live queries, and local content provided by users on their own system. These different routes are reflected in the software design (Extended Data Fig. 3a–c) and result in three alternative ways for expanding the Multilevel Human Brain Atlas.

**Extended Data Fig. 5:**
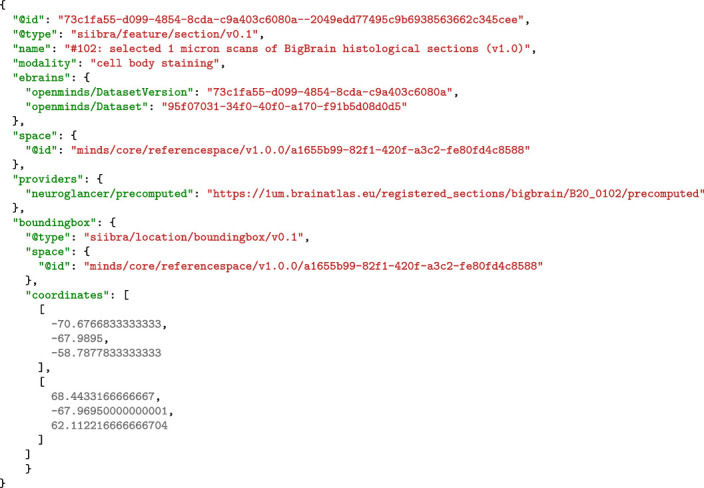
Specification of a typical image data feature. The specification is given as a json file and describes a 2D section anchored to the BigBrain reference space. The original file is part of siibra’s default configuration available at github. It follows the schema ‘section/v0.1’ defining text fields for dataset name and modality, as well as URLs to relevant openMINDS metadata entities of the corresponding dataset in EBRAINS. The actual content is identified by the URL to the multi-resolution image data in neuroglancer precomputed format, and associated with the BigBrain reference space using a unique identifier. Using this information, siibra is able to extract detailed metadata and access the full resolution image data as demonstrated in Figs. 2d-f and 5e.

First, new foundational contents are added to siibra’s configuration by creating specification files (Extended Data Fig. 5). This allows flexible addition of different object types from any accessible data resource. For adding specifications to the default configuration of the Multilevel Human Brain Atlas, a change request can be submitted at https://github.com/FZJ-INM1-BDA/siibra-configurations. Local installations of siibra-python can use custom configuration repositories, which allow to clone an existing configuration to a local computer add new specification files directly.

Second, dynamic content can be made discoverable in siibra live queries by sharing it on a supported cloud service. For example, when releasing a public dataset on EBRAINS, information about its anatomical location will be modeled according to the openMINDS SANDS metadata model (RRID:SCR_023498) and stored in the EBRAINS Knowledge Graph (RRID:SCR_017612). This allows siibra’s EBRAINS live query to match it with atlas elements and include it automatically. To connect new cloud services, siibra-python can be extended with new live queries.

Finally, local content can be added ad-hoc in siibra-python using various utility functions (for example, Fig. 5c), including both reference atlases and data features. For example, users may add a custom parcellation map or an image volume to query data features, demonstrated for the AICHA atlas^29^ in the code example available from ref. ^30^. In siibra-explorer, user-supplied NIfTI volumes can be visualized on top of existing reference atlases by drag-and-drop onto the browser window, as illustrated in a video tutorial included in the documentation at https://siibra-explorer.readthedocs.io (ref. ^21^). Assuming it has been prealigned to the respective reference coordinate space, the file is co-visualized in the browser while only remaining on the user’s local system.

**Extended Data Fig. 6:**
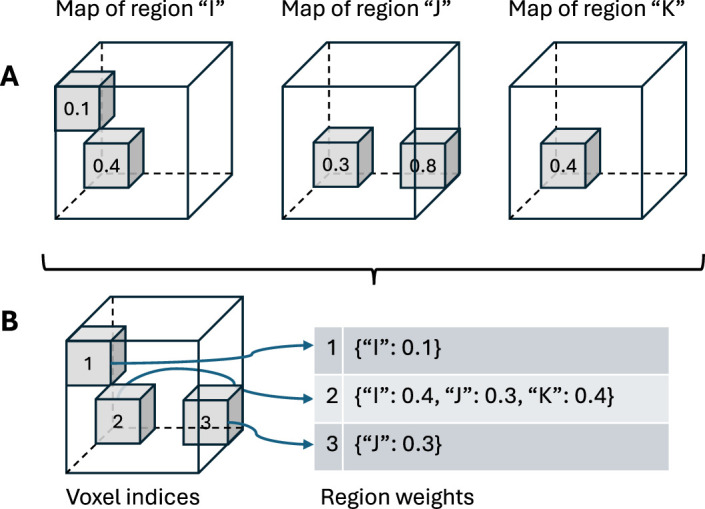
Representation of sparse statistical parcellation maps used in siibra. (**a**) Three hypothetical statistical maps for different brain areas ‘I’, ‘J’ and ‘K’, represented as simplistic arrays of 3 × 3 × 3 voxels with only a few nonzero weights each. (**b**) The statistical maps are converted into one single array storing indices to all ‘occupied’ voxel coordinates, together with a list of the map weights associated with these voxels. The weights for a given voxel are stored in the list as a dictionary which maps from the corresponding brain area to the weight.

In some cases, it can be advisable to create and publish a derived dataset when adding new content to siibra. A common example is to convert a high-resolution image into a multi-resolution format for more efficient access, or to convert a multi-volume parcellation map into a single volume with a lookup table for more efficient assignment of brain areas (Extended Data Fig. 6).

In addition to the Multilevel Human Brain Atlas presented here, siibra can be used for brains of other species without advanced programming skills. It has been successfully applied to set up the macaque brain atlas^31–33^ available at https://atlases.ebrains.eu/viewer/go/mebrains, the Waxholm Space Atlas of the Sprague Dawley Rat Brain^34–37^ available at https://atlases.ebrains.eu/viewer/go/rat, and the Allen Mouse Brain Atlas^38^ (https://atlases.ebrains.eu/viewer/go/mouse). A minimal configuration for a new atlas requires three json specifications, namely (1) a terminology of brain areas, (2) a reference coordinate system with an anatomical template in volume or surface form, and (3) a parcellation map in volume or surface form defined in the same coordinate system. This implies to host at least two image volumes or surface meshes on a web-accessible resource.

## Discussion

The development of a Multilevel Human Brain Atlas covering the scales from cells and fibers up to the macrostructure of the entire organ requires novel conceptual and technical solutions. To exploit the full potential of large-scale, high-resolution measurements from modern imaging techniques, the operationalization of such a system inevitably requires instruments for handling big data resources.

siibra facilitated the systematic implementation of an openly accessible and interoperable Multilevel Human Brain Atlas that links complex multimodal datasets with reference parcellations in different coordinate systems. We use cytoarchitecture as a fundamental principle of brain organization in the atlas, because it provides an adequate spatial scale and represents a common reference modality for neuroscientific findings. The use of probabilistic maps for assigning regions of interest to brain areas defined in reference atlases is encouraged, as a statistical treatment is crucial for modeling common uncertainties of in vivo analyses. The v.3.1 of the Julich-Brain reference parcellation includes 151 cortical areas and 56 subcortical structures mapped in both hemispheres, totaling 414 maps. Remaining GapMaps occupy about 25% of the cortex, each consisting of an integer number of areas where mapping is still in progress. Their size is decreasing with each release of Julich-Brain (v.2.9, 38.2%; v.3.0.3, 36.7%; v.3.1, 25.4%; upcoming v.3.2 to in 2025, 18%). The additional high-resolution full 3D maps in BigBrain space stem from a new AI-supported workflow with continuous release cycles. The current set includes here 122 maps (61 regions mapped in both hemispheres).

Being designed as a distributed system that can connect to a multitude of configurable cloud resources, siibra embraces the rapid growth in high-throughput imaging techniques and increasing availability of big data repositories. The tool suite is well integrated and supported by the EBRAINS research infrastructure, which allowed us to connect it with a broad compilation of datasets, tools and online services. At the time of writing, the Multilevel Human Brain Atlas provides access to more than 7 TB of multimodal datasets in the form of 2D and 3D images, tables and numerical arrays, but its design allows for continuous expansion with new instances of reference atlases and data features. Given our observation of variable response time depending on geographic location, it will be crucial for broad acceptance of siibra as a research tool to setup a globally distributed ecosystem of redundant cloud resources and provide well-balanced performance from different geographic locations. Possible strategies need to be explored in suitable infrastructure consortia such as EBRAINS or EuroHPC.

The siibra tool suite offers accessibility to user groups with different programming skills: non-programmers can engage interactively via a web browser, computationally adept neuroscientists can utilize scripting and workflow construction in Python, and professional programmers can leverage a web API for application development. The versatility therefore spans from neuroimaging and artificial intelligence to computational neuroscience and clinical applications. This has been demonstrated in first studies applying siibra successfully, for example for modeling whole brain dynamics with regional heterogeneity^39^ and investigating structure–function relationships^40^.

The development of the Multilevel Human Brain Atlas using siibra was closely integrated with ongoing community efforts in metadata standardization, resulting in foundational atlas content being curated according to the openMINDS metadata framework (RRID:SCR_023173), and fundamental design principles in siibra following the AtOM ontology model^9^. The current version of siibra puts a focus on foundational data resources established in EBRAINS by the Human Brain Project^41^, but it is designed from ground up to interface more broadly with a growing portfolio of neuroscience community resources. Upcoming releases of siibra will link with recently proposed repositories for collecting file-based MRI-scale reference templates and parcellations^4,42–44^ and online resources for additional data modalities such as human cell morphologies^45^ and functional measurements^46^. The integration of such data resources on the basis of siibra live queries is much facilitated by the growing adoption of the BIDS standard^47^ and its recently proposed extensions for other data modalities (for example^48^).

As big data and AI increasingly become central pillars of neuroscientific research^49,50^, we face the challenge of how to involve researchers in complex reasoning and discovery processes. The combination of big data visualization (in siibra-explorer) and systematic data processing (in siibra-python) provides new opportunities for designing visual analytics workflows^51–53^. These aim to combine purely data-driven exploration with human cognitive, perceptual and reasoning abilities by building visually supported loops of data extraction, hypothesis building, and inference. With the advent of AI and big data, visual analytics is expected to gain increasing interest in the neuroscience community in the future. Software solutions such as siibra are key enablers of such developments.

From a technical perspective, siibra covers core principles of data aggregation systems^54,55^ by addressing the collection, processing, and combination of data from multiple sources to better enable downstream tasks such as hypothesis generation, analytics, modeling and inference, and by solving problems such as redundancy, inconsistencies, and varying data formats. In brain research, addressing these informatics challenges is crucial for harnessing data in large-scale, data-driven models like AI foundation models, anatomically accurate simulations, and digital twins, paving the way for new research questions to be explored. Of course, data aggregation systems play a crucial field in other fields. Although siibra was implemented for neuroscientists in the first place, the key aspects of its implementation are not specific to this field. With moderate adaptions of the code, it could serve as a basis for building atlases of other organs such as the heart or kidney, and more generally for data aggregation of systems with a strong 3D topographical organization which are described by images and tabular content at multiple scales.

In conclusion, the siibra tool suite introduces a highly configurable informatics framework for building multilevel brain atlases of different species and organs, combining big data capabilities with broad interoperability across distributed data resources and community tools, in support of FAIR principles.

## Methods

In the following, we describe relevant methods and technical principles applied in the siibra tool suite, on which the presented results are based. These include modularization of the software, separation of code from contents and strategies that enable the use of large image datasets and locations in different reference coordinate systems.

### Modeling spatial entities in different reference coordinate systems

In line with community ideas^9,64^, siibra distinguishes semantic atlas elements, such as reference coordinate systems and terminologies, from their spatial representations as annotations sets or reference templates. This is a prerequisite for using the same anatomical concepts in processes operating at different spatial scales or topological layouts. For example, the same atlas terminology can be realized by annotation sets in different reference coordinate systems, thereby resulting in multiple representations of the same brain area with different coordinate system, spatial resolution or data format. Similarly, a reference coordinate system can be represented by different reference datasets (templates) with specific resolution and representation of the underlying anatomy as an image volume or surface.

In general, points in different reference coordinate systems are related by nonlinear coordinate transformations. This association is not unique and generally inaccurate, as different mathematical models and optimization strategies for the transformation result in different solutions. For the Multilevel Human Brain Atlas, we used diffeomorphisms constrained with cortical folding patterns^65,66^ to obtain a precise alignment between reference coordinate systems in regions where sulci can be used as landmarks. These transformations are exposed through a web service (https://github.com/HumanBrainProject/hbp-spatial-backend), which siibra uses to transform coordinates between reference coordinate systems when querying data features or comparing spatial objects.

All spatial entities in siibra-python reflect instances of locations such as points, point clouds or bounding boxes (Extended Data Fig. 3d–f). Examples of these three types of instances are centroids of brain areas, bounding boxes of volumes of interest, contact points of electrodes, peaks of fMRI activations maps, or the currently selected view in siibra-explorer. As any such location object is uniquely associated with a reference coordinate system, coordinate projections can be carried out automatically, thus allowing to apply most spatial operations in siibra safely to objects from different reference coordinate spaces. This principle has been demonstrated in the results for cortical image patch sampling, where the bounding box of a brain area in MNI space is automatically transferred to BigBrain space for finding high-resolution image data (Fig. 5) and a set of vertices in BigBrain space is evaluated by a probability map in MNI space.

The precision of cross-subject matching using these spatial transformations has been evaluated for cortex overlap and sulcal dispersion in ref. ^66^. To provide an additional assessment of their practical application for coordinate warping in siibra, we carried out two experiments, with results compiled in Extended Data Fig. 2:

1. Reprojection errors were computed when warping random gray matter positions from one template space to another and back (Extended Data Fig. 2a). The experiment was performed with *N* = 5,000 points between the ICBM 2009c nonl asym, Colin 27 and BigBrain spaces. Average reprojection errors were in the range of 10–20 μm, occasionally reaching the range of 100–200 μm. These are attributed to numerical limitations arising from the large-scale differences between the spaces and the need for serialization of the floating numbers during network communication.
2. For cytoarchitectonic brain areas mapped in BigBrain, 500 random locations each were sampled uniformly from the ultrahigh-resolution 3D BigBrain map, and warped to the ICBM 2009c asymmetric space. Their probability to lie consistently inside the same brain area was then determined in the target space using the corresponding Julich-Brain probabilistic map (Extended Data Fig. 2c). Except for occasional outliers close to the areal borders, warped points were located at nonzero probabilities for all evaluated brain areas, whereas the average probability score varied substantially between the regions. This can be expected, because the probability maps in MNI space capture the variance of mappings from ten different postmortem brains^6^ after spatial normalization, and the individual subject map in BigBrain space does not necessarily coincide with their average.

### Linking data features to atlas elements

The localization of data features with respect to atlas elements can take various forms with different levels of precision. In a human brain atlas, this is particularly influenced by the high inter-subject variability, resulting in potentially considerable uncertainties when relating spatial locations measured in different brains. The most accurate localization is possible for measurements obtained in the same brain, where for example image registration can provide adequate alignments. For different brains, an accurate correspondence can be achieved if the data allow reliable histological identification of the underlying brain area. This principle is used extensively for the Multilevel Human Brain Atlas: cytoarchitectonic maps are being used as a preferred reference to localize foundational data features such as neurotransmitter receptor and cell densities according to cytoarchitectonic criteria of the tissue samples. This provides an intrinsic definition of anatomical locations, directly grounded in properties contained in the measurement, and therefore independently verifiable. For other datasets such as in vivo neuroimaging or physiological recordings, the assignment often has to rely on projections of coordinates. This can be seen as an extrinsic transfer of the localization to the data, which is not uniquely defined and less verifiable.

To capture different forms of anatomical correspondence between atlas elements and data features, siibra tags anatomical locations with semantic relationships to brain areas from a reference parcellation, with spatial locations such as points, lines or bounding boxes in a reference coordinate space, or combinations thereof. As such it implements basic principles of the Locare workflow proposed for representing locations in 3D brain atlases^67^. The semantic or spatial location specifications can be further refined by certainty quantifications such as information about containedness and overlap of brain structures, accuracy of coordinates or hierarchical dependencies between child and parent areas in an atlas terminology.

As a result, siibra can query many forms of data features in a unified fashion by providing an anatomical concept (such as a brain area or volume of interest) and a modality for the requested data features. When evaluating such a query, siibra uses all available information to compute a qualification for each matched data feature. This enables an adequate assessment of the relevance and accuracy of retrieved measurements with regard to a given analysis task. A practical example of this principle can be reproduced using the notebook available at https://siibra-python.readthedocs.io/en/v1/examples/03_data_features/000_matchings.html.

### Modular software architecture

The siibra tool suite consists of an interactive 3D web viewer (siibra-explorer), an HTTP API (siibra-api) and a Python library (siibra-python). A command line client and MATLAB library (https://github.com/FZJ-INM1-BDA/siibralab) are also being developed, but are not covered in this contribution. Following a modular software design, these components share most of their functionality while addressing different user scenarios. The core functionality is implemented in siibra-python, a Python package suitable for scripting, interactive notebooks and development of computational workflows. The key elements of the system architecture are illustrated in Extended Data Fig. 3. It models various elements of reference atlases, coordinate-based locations, data features and queries; and provides functionalities for loading, matching and processing these entities. As the contents are managed separately from the software, efficient retrieval of data from cloud resources becomes an essential aspect of the package. Various data-fetching mechanisms are implemented for files, multi-resolution images and metadata, following a lazy loading strategy to ensure that only actually requested elements are transferred. The package documentation is hosted at https://siibra-python.readthedocs.io and automatically updated with new releases. It contains a comprehensive collection of documented and tested code examples that demonstrate typical use cases, including the executable codes to reproduce the figures in our results. These are complemented by a growing collection of more comprehensive tutorial notebooks available at https://github.com/FZJ-INM1-BDA/siibra-tutorials, developed for workshops and user training.

The functionalities of siibra-python are exposed as a RESTful API for application development (siibra-api). This HTTP interface focuses on the processing of HTTP requests and efficient serialization of siibra objects and method calls using FastAPI (https://fastapi.tiangolo.com) as a web framework, and implements type-hinting via OpenAPI (https://spec.openapis.org/oas/latest.html). A production instance is deployed, documented and openly accessible for application developers at https://siibra-api.apps.ebrains.eu/v3_0/docs.

Finally, the web viewer siibra-explorer implements an interactive user interface on top of siibra-api, focusing on visualization and user interactions while reusing the underlying functionalities from siibra-python. It uses Angular (angular.dev) as a reactive programming framework, neuroglancer^68^ as a visualization backbone for very large multiresolution volumetric images, and ThreeJS (threejs.org) for rendering brain surfaces. The surface view is automatically activated when selecting the Freesurfer space, and provides white matter, pial and inflated surface representations. The viewer can be accessed from common web browsers, including mobile devices with touch inputs such as iOS or Android tablets.

The production instance of the viewer provides access to the Multilevel Human Brain Atlas presented in this paper and is available at https://atlases.ebrains.eu/viewer. To track the community interest in the atlas, we record the numbers of unique returning visitors per month on this instance, which has been increasing over a timescale of 4 years from about 400 (summer 2020) to about 1,300 (summer 2024).

Documentation for siibra-explorer is hosted at https://siibra-explorer.readthedocs.io. It provides a guided quick tour to help users to get started, which is automatically launched on first use and can later be accessed via the help menu.

To keep the development of siibra-explorer focused on its core requirements while still allowing flexible addition of functionalities, the viewer supports the implementation of plugins similar to the add-on mechanisms offered by modern web browsers. This has been used to create an interactive implementation of the JuGEx workflow^69^, which can be accessed from the plugin menu when selecting the MNI 152 reference space. The plugin architecture has also been used successfully by researchers to integrate a workflow for learning gene regulatory networks with Bayesian networks^70^.

### Separation of content from code

A fundamental design principle of siibra is separation of content from software, which is an important prerequisite for extensibility.

#### Specification files

The foundational content elements such as parcellation maps, reference templates and data features are connected to siibra through specification files maintained outside the codebase. These specifications follow a set of simple schemata maintained in the config_schema folder of siibra-python. Typically, a specification file defines (1) minimal metadata entries such as identifier, name, and modality, (2) pointers to an external metadata management system such as the EBRAINS Knowledge Graph providing extended metadata, (3) an anatomical reference such as a coordinate-based location or a brain area name from a reference atlas, and (4) URLs and format specifiers that identify the underlying data, such as image or tabular data files stored on a cloud resource. The specification of a basic image data feature, taken from the configuration at https://github.com/FZJ-INM1-BDA/siibra-configurations/tree/v1/features/images/sections/cellbody, is displayed in Extended Data Fig. 5. While some specifications, such as for parcellation maps, are more comprehensive, it shows the typical style and organization of the format. A more complete documentation of siibra specifications is available as part of the code at https://github.com/FZJ-INM1-BDA/siibra-python/tree/v1/config_schema.

#### Configurations

Foundational content specifications are collected into configurations by packaging json files into a zip container, git repository, or folder on a file system. Configurations can be hosted both on a local machine or on a cloud service. The default configuration of siibra provides the foundational content of the Multilevel Human Brain Atlas. It is maintained at https://github.com/FZJ-INM1-BDA/siibra-configurations, continuously updated by the maintainers and open for extension requests by the community. Local installations of siibra can use alternative or extended configuration repositories with customized content, ensuring well-defined maintenance, extension and customization of atlas contents.

#### Live queries

Foundational content specified in configurations is extended by dynamic content from siibra live queries (software interfaces implemented in siibra-python for connecting to APIs of suitable cloud services and translating their responses into siibra specifications at runtime). Implementing live queries assumes external repositories to provide a stable API and to return sufficient metadata and common access URLs to generate specifications as described above. So far, siibra includes live queries to the EBRAINS Knowledge Graph (RRID:SCR_017612) for discovering anatomically anchored datasets, to the Allen Human Brain Atlas^18,19^ for accessing microarray data localized in MNI space, and to cloud image resources, for example^28^, for sampling high-resolution image data as demonstrated in Fig. 5.

### Support of FAIR principles

Most foundational content in siibra’s default configuration points to curated datasets in EBRAINS, supporting the FAIR principles for data sharing^71^. Registration in the EBRAINS Knowledge Graph (https://docs.kg.ebrains.eu) (RRID:SCR_017612) ensures findability, as it provides each content element an individual DOI, links it with rich metadata and offers comprehensive faceted search interfaces. The search paths offered by the Knowledge Graph are further complemented by siibra in terms of more advanced anatomical navigation and atlas-guided processing. Accessibility of rich metadata is provided for atlas content through their DOIs, which are exposed by siibra and allow direct online access to original dataset pages including comprehensive data descriptors. The siibra tool suite adds additional entry points for retrieval by forwarding the metadata, offering download links in siibra-explorer, and implementing efficient data-fetching capabilities in siibra-python. By promoting the openMINDS metadata framework(RRID:SCR_023173) used by the EBRAINS Knowledge Graph (RRID:SCR_017612), siibra further supports interoperability and reusability. Instead of establishing a different knowledge representation, siibra specification schemas only serve as a summary and reference pointer to the established representations and terminologies in EBRAINS. By explicitly subjecting the content of each foundational atlas element to the professional data curation offered by EBRAINS, a comprehensive description of the content with attributes accepted by the community is ensured. Interoperability is further optimized by exposing the retrieved contents from the underlying dataset in highly interoperable formats. For example, in siibra-python, fetched image data are exposed as Nifti1Image image objects via NiBabel (RRID:SCR_002498), fetched tabular data as pandas (RRID:SCR_018214) DataFrames, and more general numerical data, such as vertex lists, as NumPy (RRID:SCR_008633) arrays (Fig. 3). This makes it straightforward to use the data with popular tools such as Nilearn (RRID:SCR_001362), ITK-SNAP (www.itksnap.org), napari (RRID:SCR_022765) and Matplotlib (RRID:SCR_008624) and integrate it in common analysis workflows.

### Unified access to small and big image volumes

A central design goal of siibra is to unify access to small and ‘big’ image data, for example to handle images in the range of a few hundred megabytes up to terabyte scale in a consistent fashion while hiding the complexity of their different access paths as cloud volumes or downloadable files. This has been realized by supporting both conventional local and web-hosted NIfTI files as well as multi-resolution chunk formats such as the neuroglancer precomputed tile format through the same user-level functionality. The heterogeneity of these data types is hidden behind a common interface in siibra-python, which provides image data in NIfTI format with appropriate spatial metadata independently of its original format and origin. When accessing content elements with conventional ‘small’ 3D image data, siibra will load and forward the original image data in unmodified form. When fetching from a large multi-resolution image resource, siibra will either return an appropriately downscaled version or require us to specify a region of interest that results in a feasible download size (Fig. 5e). In any case, the involved cropping or scaling will be reflected in the spatial metadata of the resulting NIfTI object, so that the fetched data remain appropriately localized in their original reference space. This way, siibra can convert large whole-brain image resources stored on cloud resources into actionable data structures as required by the user. This principle can be reproduced using the notebook available in the software documentation in ref. ^72^.

### Memory-efficient representation of sparse regional maps

Statistical maps associate 3D points in a reference coordinate space with continuous weights reflecting the likelihood of a brain area being present at that location^64^. Processing such statistical parcellation maps, for example for probabilistic assignment (Fig. 3), thus might imply retrieving and loading one image volume per brain area. For Julich-Brain, this amounts to loading approximately 300 3D volumes in MNI space, which results in long download times for the initial data fetching and may quickly exceed the working memory capacity of standard computers. To avoid these problems, siibra implements an alternative data structure to minimize memory consumption. The structure exploits the fact that statistical region maps often encode only a small fraction of the brain volume, thus mostly containing zeros and effectively forming an extremely sparse four-dimensional array over all brain areas. The main idea is to replace these sparsely populated 3D volumes with an index that stores only nonzero weights. This is modeled by two data structures, which may be understood as a ‘spatial’ dictionary with coordinate-based keys (Extended Data Fig. 6): The first structure is a single 3D array matching the original voxel dimensions of the maps. This array structure allows efficient spatial queries with bounding boxes or image masks. Elements of the array are zero if the corresponding voxel is not associated with any brain area in the parcellation, or hold a numerical key otherwise. The numerical keys are indices to the second structure, a list containing the statistical weights of the corresponding voxel statistical maps concerned. The structure reimplements all functions of conventional parcellation maps, including data fetching and assignment of coordinates to brain areas.

### Additional software and visualization

Processing and visualization were performed in Python 3. Tabular data were processed using pandas^73^. Figures were generated using matplotlib^74^ and seaborn^75^. Neuroimaging visualizations were created using Nilearn^76^.

### Reporting summary

Further information on research design is available in the Nature Portfolio Reporting Summary linked to this article.

## Online content

Any methods, additional references, Nature Portfolio reporting summaries, source data, extended data, supplementary information, acknowledgements, peer review information; details of author contributions and competing interests; and statements of data and code availability are available at 10.1038/s41592-026-03159-x.

## Supplementary information

Reporting Summary

Peer Review File

## Figures and Tables

**Extended Data Table 1:**
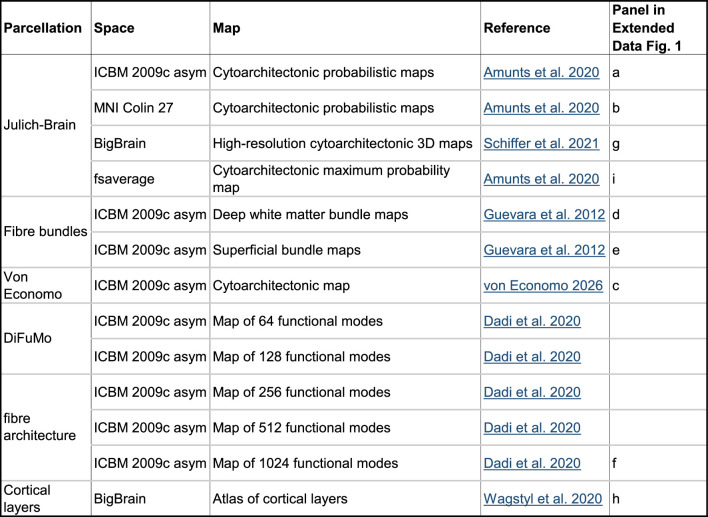
Reference parcellations in the current configuration of the Multilevel Human Brain Atlas

Set of parcellation maps included in default siibra configuration for the Multilevel Human Brain Atlas at the time of writing. The reported version of the configuration is available at github. The system is designed to be extended with more parcellations and versions. Local installations of siibra further allow to use configurations with custom maps. A selection of the parcellations are displayed in Extended Data Fig. 1, as specified in the rightmost column.

**Extended Data Table 2:**
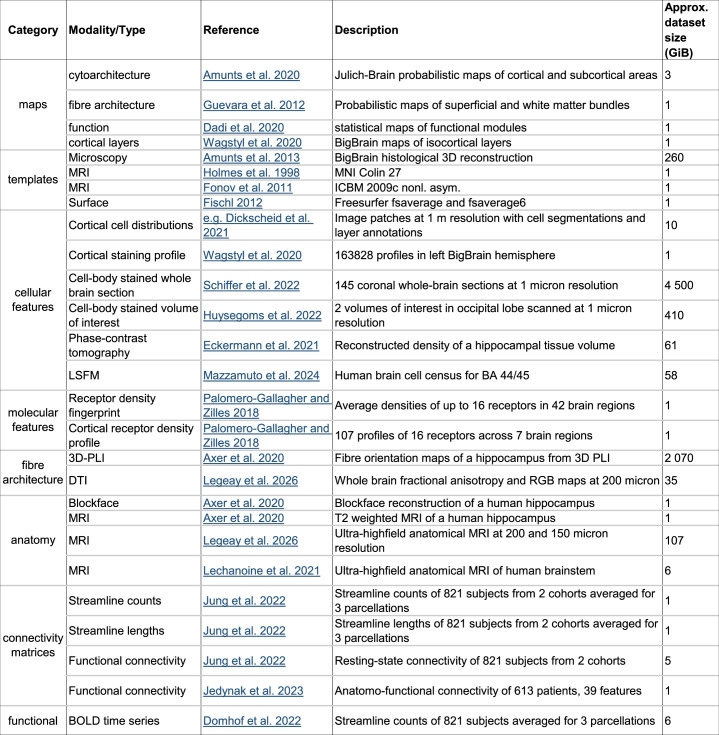
Snapshot of foundational content in the Multilevel Human Brain Atlas

Set of foundational data specified in the default siibra configuration of the Multilevel Human Brain Atlas at the time of writing, including maps, templates and multimodal data features. The amount of data features that can actually be accessed is larger due to dynamic content obtained through live queries as well as continuous extension of the foundational content in siibra’s default configuration. The reported version of the configuration is available at github.

## Data Availability

All data used in this work are publicly available via the URLs and references provided with the respective figures. Most contents are published as curated datasets with permanent identifiers on the EBRAINS platform (https://ebrains.eu). Gene expression levels were retrieved from the Allen Human Brain microarray data^18,19^. The current foundational content linked with the atlas is defined and maintained at https://github.com/FZJ-INM1-BDA/siibra-configurations/tree/v1. An overview is provided as Extended Data Table 2.

## Code availability

Source codes for siibra are available on GitHub (https://github.com/FZJ-INM1-BDA/siibra-python; https://github.com/FZJ-INM1-BDA/siibra-explorer and https://github.com/FZJ-INM1-BDA/siibra-api) and provided under the Apache 2.0 license (http://www.apache.org/licenses/). We have integrated all components of siibra with Zenodo, which generates unique DOIs for each new release of the tool suite. An installation of the interactive viewer is publicly accessible at https://atlases.ebrains.eu/viewer as a core component of the brain atlas services in the research infrastructure EBRAINS. The web API can be accessed at https://siibra-api.apps.ebrains.eu/v3_0/docs. Researchers can install siibra-python through the Python Package Index from https://pypi.org/project/siibra. Comprehensive online documentation, including executable codes to reproduce all figures can be accessed via https://siibra-python.readthedocs.io/en/v1. More detailed tutorials notebooks are provided at https://github.com/FZJ-INM1-BDA/siibra-tutorials.
