# Supplementary material for: Siibra: a software tool suite for realizing a Multilevel Human Brain Atlas from complex data resources
Source: Nat Methods. 2026 Jul 20;23(8):1647–58. doi: 10.1038/s41592-026-03159-x (PMC13441889; doi:10.1038/s41592-026-03159-x)
Supplement: Supplementary file 1 [file 41592_2026_3159_MOESM1_ESM.pdf]

nature portfolio

Corresponding author(s): Timo Dickscheid, Katrin Amunts

Last updated by author(s): Apr 27, 2026

Reporting Summary

Nature Portfolio wishes to improve the reproducibility of the work that we publish. This form provides structure for consistency and transparency in reporting. For further information on Nature Portfolio policies, see our Editorial Policies and the Editorial Policy Checklist.

Statistics

For all statistical analyses, confirm that the following items are present in the figure legend, table legend, main text, or Methods section.

n/a	Confirmed	
☐	☒	The exact sample size (*n*) for each experimental group/condition, given as a discrete number and unit of measurement
☐	☒	A statement on whether measurements were taken from distinct samples or whether the same sample was measured repeatedly
☒	☐	The statistical test(s) used AND whether they are one- or two-sided<br>*Only common tests should be described solely by name; describe more complex techniques in the Methods section.*
☒	☐	A description of all covariates tested
☒	☐	A description of any assumptions or corrections, such as tests of normality and adjustment for multiple comparisons
☒	☐	A full description of the statistical parameters including central tendency (e.g. means) or other basic estimates (e.g. regression coefficient) AND variation (e.g. standard deviation) or associated estimates of uncertainty (e.g. confidence intervals)
☒	☐	For null hypothesis testing, the test statistic (e.g. *F*, *t*, *r*) with confidence intervals, effect sizes, degrees of freedom and *P* value noted<br>*Give P values as exact values whenever suitable.*
☒	☐	For Bayesian analysis, information on the choice of priors and Markov chain Monte Carlo settings
☒	☐	For hierarchical and complex designs, identification of the appropriate level for tests and full reporting of outcomes
☒	☐	Estimates of effect sizes (e.g. Cohen's *d*, Pearson's *r*), indicating how they were calculated

Our web collection on statistics for biologists contains articles on many of the points above.

Software and code

Policy information about availability of computer code

Data collection	Data shown in this study has been retrieved using siibra-python v1.0.1 and siibra-explorer 2.14.17. The software codes are openly accessible on github through the URLs provided in the manuscript. We provide the codes to reproduce the content of each figure as publicly available executable Jupyter notebooks, with URLs provided in the figure legends. A list of lower level software libraries used internally by siibra is available through the file requirements.txt in the main folder of each github repository (e.g. https://github.com/FZJ-INM1-BDA/siibra-python).
Data analysis	The manuscript does not involve advanced data analyses beyond fetching, formatting and plotting data from previously published datasets. In Fig. 4 and Fig. 5, plotting involved to display mean, median, percentiles and ranges of a column in a fetched data table using the functions provided by matplotlib and pandas. Anatomical assignment of example image data to brain regions in Figs. 3+4 was performed using siibra-python v1.0.1 and can be reproduced with openly available Jupyter notebooks, available on github.com and readthedocs.io using the URLs provided in the Figure legends.

For manuscripts utilizing custom algorithms or software that are central to the research but not yet described in published literature, software must be made available to editors and reviewers. We strongly encourage code deposition in a community repository (e.g. GitHub). See the Nature Portfolio guidelines for submitting code & software for further information.

Data

Policy information about availability of data

All manuscripts must include a data availability statement. This statement should provide the following information, where applicable:

- Accession codes, unique identifiers, or web links for publicly available datasets
- A description of any restrictions on data availability
- For clinical datasets or third party data, please ensure that the statement adheres to our policy

All data presented in the manuscript are publicly available via the URLs and references provided with the respective figures, and originate from previously published EBRAINS datasets with the exception of the gene expression data which originates from the Allen Human Brain Microarray database referenced in the manuscript. An overview of all data is provided in Extended Data Table 2.

Research involving human participants, their data, or biological material

Policy information about studies with human participants or human data. See also policy information about sex, gender (identity/presentation), and sexual orientation and race, ethnicity and racism.

Reporting on sex and gender	Not applicable. The manuscript addresses processing of existing neuroscience datasets via anatomical locations in the brain, such as specified coordinates, coordinate ranges, or names of brain areas from a reference atlas. For sex and gender information we refer to the data descriptors available with each dataset on their original resource. For example, users of the siibra toolsuite can access sex- and gender specifications as provided in the metadata of each collected dataset; in most cases modelled according to the openMINDS metadata framework in EBRAINS.
Reporting on race, ethnicity, or other socially relevant groupings	Not applicable. The manuscript does not make any use of any of these groupings. Users of siibra will be able to access such attributes from the metadata of the underlying datasets. The availability of such attributes depends on each underlying dataset and is not part of the siibra tool suite.
Population characteristics	Not applicable. No human subjects were recruited for this study. The connectivity profiles displayed in Figs. 3+4 originate from datasets that were derived from human imaging cohorts, in particular the Human Connectome Project and the 1000 brains study. References are given for these two repositories. Information about the underlying population characteristics is contained in the data descriptors of these datasets, available through the dataset DOIs listed in the manuscript. The manuscript does not use these characteristics, it only displays a global average for purposes of illustration.
Recruitment	Not applicable. No participants were recruited for this study.
Ethics oversight	The present manuscript did not require approval of a study protocol.

Note that full information on the approval of the study protocol must also be provided in the manuscript.

Field-specific reporting

Please select the one below that is the best fit for your research. If you are not sure, read the appropriate sections before making your selection.

☒ Life sciences ☐ Behavioural & social sciences ☐ Ecological, evolutionary & environmental sciences

For a reference copy of the document with all sections, see nature.com/documents/nr-reporting-summary-flat.pdf

Life sciences study design

All studies must disclose on these points even when the disclosure is negative.

Sample size	The manuscript does not involve statistical analyses that would require a calculation of sample sizes.
Data exclusions	The manuscript does not involve statistical analyses for which data was excluded.
Replication	The manuscript does not present experimental findings, since it focuses on demonstrating how information can be assembled using the brain atlas. All figures illustrating data collection can be reproduced using corresponding Python notebooks which we share as part of the software documentation. These notebooks are built automatically and verified to produce the outputs included in the manuscript. On publication of the manuscript, we will also include this verification as part of the default testing procedure.
Randomization	The manuscript does not involve statistical analyses for which allocation of samples into experimental groups is relevant.
Blinding	The manuscript does not involve statistical analyses for which blinding for group allocation of samples is relevant.

Reporting for specific materials, systems and methods

We require information from authors about some types of materials, experimental systems and methods used in many studies. Here, indicate whether each material, system or method listed is relevant to your study. If you are not sure if a list item applies to your research, read the appropriate section before selecting a response.

Materials & experimental systems

n/a	Involved in the study
☒	☐ Antibodies
☒	☐ Eukaryotic cell lines
☒	☐ Palaeontology and archaeology
☒	☐ Animals and other organisms
☒	☐ Clinical data
☒	☐ Dual use research of concern
☒	☐ Plants

Methods

n/a	Involved in the study
☒	☐ ChIP-seq
☒	☐ Flow cytometry
☒	☐ MRI-based neuroimaging

Plants

Seed stocks	Not applicable.
Novel plant genotypes	Not applicable.
Authentication	Not applicable.
