# Supplementary material for: Siibra: a software tool suite for realizing a Multilevel Human Brain Atlas from complex data resources
Source: Nat Methods. 2026 Jul 20;23(8):1647–58. doi: 10.1038/s41592-026-03159-x (PMC13441889; doi:10.1038/s41592-026-03159-x)
Supplement: Supplementary file 2 [file 41592_2026_3159_MOESM2_ESM.pdf]

nature portfolio

Peer Review File

Siibra: A software tool suite for realizing a Multilevel Human Brain Atlas from complex data resources

Corresponding Author: Professor Timo Dickscheid

This file contains all editorial decision letters in order by version, followed by all author rebuttals in order by version.

Version 0:

Decision Letter:

3rd Jul 2025

Dear Professor Dickscheid,

Let me first apologize for the delay in the review process. Your Article entitled "Siibra: A software tool suite for realizing a Multilevel Human Brain Atlas from complex data resources" has now been seen by two reviewers, whose comments are attached. While they find your work of potential interest, they have raised serious concerns which in our view are sufficiently important that they preclude publication of the work in Nature Methods, at least in its present form.

As you will see, the reviewers raise concerns about utility of the platform, with respect to the available atlases as well as performance of the web platform. Related to the latter point, the reviewers also ask for further validation.

Should further experimental data allow you to fully address these criticisms we would be willing to look at a revised manuscript (unless, of course, something similar has by then been accepted at Nature Methods or appeared elsewhere). This includes submission or publication of a portion of this work somewhere else. We hope you understand that until we have read the revised paper in its entirety we cannot promise that it will be sent back for peer-review.

If you are interested in revising this manuscript for submission to Nature Methods in the future, please contact me to discuss your appeal before making any revisions. Otherwise, we hope that you find the reviewers' comments helpful when preparing your paper for submission elsewhere.

Best regards,
Nina

Nina Vogt, PhD
Senior Editor
Nature Methods

Reviewers' Comments:

Reviewer #1:
Remarks to the Author:
Dickscheid et al. present "siibra" (Software Interfaces for Interacting with BRain Atlases), a comprehensive open science software suite designed to support the development of a multimodal human brain atlas by integrating diverse multiscale data resources into a coherent anatomical template. It offers a web viewer, a Python library, and an HTTP API, aiming to facilitate access from macroscopic structures down to cellular details. The authors accurately identify and address key challenges involved in multimodal data integration, including interoperability, reproducibility, and scalability. Siibra effectively leverages cloud resources from the EBRAINS infrastructure and other repositories, providing efficient data retrieval and visualization for interactive exploration, computational analysis, and application development. The manuscript systematically guides readers through the extensive features of siibra, which is indeed necessary given the complexity of the data and potential applications.

Despite the impressive work underlying siibra and its fantastic potential utility, my hands-on experience revealed significant

practical limitations.

Specific Comments:
1. The manuscript claims that siibra "combines complementary reference atlases reflecting different principles of brain organization." However, I was expecting to find additional well-established cytoarchitectonic atlases, such as those by Paxinos and Mai. Instead, siibra users currently explore neuroanatomy primarily through the Jülich atlas, which by no means represents a consensus standard. This atlas predominantly covers the cerebral cortex and a limited number of subcortical regions. Thus, it cannot be considered a comprehensive neuroanatomical reference. Moreover, for most templates, the parcellation appears incomplete, which limits the software's practical utility. A more comprehensive discussion of this aspect is needed.

2. Testing a specific workflow, I selected area 4 (primary motor cortex) and attempted to switch from MNI Colin27 (JulichBrain 2.9) to the BigBrain template (same atlas). Contrary to the authors' statements, siibra did not maintain the anatomical location. Instead of remaining focused on area 4, the viewer unexpectedly shifted to area 6d. Using the cortical layer atlas of BigBrain resulted in better anatomical alignment, but the zoom level was lost during the transition. Thus, the described "precomputed nonlinear spatial transformations" did not effectively preserve location and zoom levels. Quantitative benchmarks demonstrating performance and scalability would greatly reinforce claims about siibra's ability to manage large-scale data.

3. When exploring the precentral gyrus, no anatomical area was interactively highlighted. The structural list further indicated that Areas 4a and 4p were "not mapped in this template space," raising concerns about completeness and consistency of mappings within siibra. Additionally, a dedicated section explicitly discussing limitations or potential barriers to widespread adoption, such as network dependency, hardware requirements, or integration of novel data types, is notably missing and would significantly enhance the manuscript.
4. I also question the anatomical validity of representing layer 4 in the agranular primary motor cortex. Maybe the authors referred to a paper by Helen Barbas on the apparent quasi acellular gap between layers 3 and 5, which is likely where many thalamocortical projections terminate.

5. In attempting to reproduce Figure 2's high-resolution cellular detail, after more than 10 minutes, the displayed image remained pixelated, and the promised cellular resolution was not achievable in practice.

6. During my second test (Multimodal comparison of brain areas), selecting area 44 via the search menu (MNI Colin 27 + JB v3.1) inexplicably navigated to area 3b instead. Given my limited experience with Python, I did not test data retrieval via scripts. However, if this retrieval mechanism functions as demonstrated in Figure 4, it would indeed represent a powerful and valuable feature of siibra.

In conclusion, while siibra represents an ambitious and theoretically powerful solution for neuroanatomical exploration, these significant practical issues, along with gaps in benchmarking, comparative analysis, and explicit discussion of limitations, currently limit its utility and accessibility. Addressing these concerns would substantially enhance the effectiveness, reliability, and clarity of this promising tool.

Reviewer #2:
Remarks to the Author:
Summary of Key Results: The manuscript presents Siibra, a multiscale neuroinformatics platform designed to integrate diverse human brain datasets into a harmonized atlas framework. It supports multimodal analyses by enabling spatial alignment of molecular, anatomical, and functional data across reference templates, with open-access implementation and adherence to FAIR principles.

Originality and Significance: The work is technically original and addresses a critical infrastructure gap in multilevel brain data integration. While atlas-based frameworks exist, Siibra's emphasis on interoperability, extensibility, and integration of high-resolution modalities (e.g., BigBrain histology) makes it a meaningful advance in open neuroinformatics.

Data and Methodology: The platform leverages well-structured metadata standards (e.g., openMINDS), modular architecture, and cloud-based delivery mechanisms. While these aspects are well-documented, the manuscript would benefit from a clearer validation framework to assess anatomical congruency and transformation fidelity. The lack of a demonstrative analysis case limits the methodological impact.

Statistics and Uncertainties: The manuscript is not heavily reliant on statistical modeling, but performance evaluations (e.g., query latency, system scalability) would fall under this rubric. At present, such benchmarks are not reported, leaving the platform's operational robustness largely qualitative.

Conclusions: The conclusions are consistent with the platform's described architecture but could be more robust if supported by a fully developed analytic use case demonstrating real-world application and data-driven inference.

Suggested Improvements:

Include performance metrics illustrating scalability (e.g., latency, rendering load).

Add a brief but concrete validation strategy across spatial transformations.

Demonstrate a use case illustrating the tool's analytical value.

Clarify how users can integrate custom datasets into the existing framework.

References are generally appropriate and include citations to major resources (e.g., BigBrain, Julich-Brain, EBRAINS). The inclusion of direct comparisons to other atlas-based tools would further contextualize novelty.

Clarity and Context: The manuscript is clearly written, with an informative abstract and appropriate structure. Minor improvements include:

Clarifying units in Supplementary Figure S1 (e.g., "1 pm" likely meant as 1 μm)

Specifying default template/parcellation versions in siibra-python

Including a schematic of metadata relationships to assist new users

Remarks on code availability:
Each of the three repositories—siibra-python, siibra-explorer, and siibra-api—includes a comprehensive README file with installation instructions, usage examples, and links to further documentation.

siibra-python is pip-installable and includes tested examples via ReadTheDocs. It supports reproducible workflows through versioned dependencies and continuous integration across Python 3.7–3.12 environments.

siibra-explorer provides a browser-based 3D viewer with clear developer instructions for local deployment using Node.js and Python backends. The README and environment setup files are sufficient for reproducing the frontend and backend locally.

siibra-api offers RESTful access to siibra-python functionality. While still labeled as experimental, it includes Docker-based deployment options and OpenAPI documentation for endpoint testing.

While I did not independently install and run the full stack, the documentation and modular design suggest that the results described in the manuscript are reproducible with reasonable effort.

**

Although we cannot publish your paper in its current form, it may be appropriate for another journal in the Nature Portfolio. If you wish to explore the journals and transfer your manuscript please use our manuscript transfer portal. You will not have to re-supply manuscript metadata and files, unless you wish to make modifications. For more information, please see our manuscript transfer FAQ page.

** For Nature Portfolio general information and news for authors, see http://npg.nature.com/authors.

Version 1:

Decision Letter:

Our ref: NMETH-A60247A-Z

3rd Apr 2026

Dear Timo,

Thank you for submitting your revised manuscript "Siibra: A software tool suite for realizing a Multilevel Human Brain Atlas from complex data resources" (NMETH-A60247A-Z), as well as for your patience. I was waiting to hear back from one more reviewer, but the review has not materialized. The manuscript has now been seen by one the original referees and their comments are below. The reviewer finds that the paper has improved in revision, and therefore we'll be happy in principle to publish it in Nature Methods, pending minor revisions to comply with our editorial and formatting guidelines.

We are now performing detailed checks on your paper and will send you a checklist detailing our editorial and formatting requirements within two weeks or so. Please do not upload the final materials and make any revisions until you receive this additional information from us.

TRANSPARENT PEER REVIEW
Nature Methods offers a transparent peer review option for new original research manuscripts. We encourage increased

transparency in peer review by publishing the reviewer comments, author rebuttal letters and editorial decision letters if the authors agree. Such peer review material is made available as a supplementary peer review file. **Please state in the cover letter 'I wish to participate in transparent peer review' if you want to opt in, or 'I do not wish to participate in transparent peer review' if you don't.** Failure to state your preference will result in delays in accepting your manuscript for publication.

Please note: we allow redactions to authors' rebuttal and reviewer comments in the interest of confidentiality. If you are concerned about the release of confidential data, please let us know specifically what information you would like to have removed. Please note that we cannot incorporate redactions for any other reasons. Reviewer names will be published in the peer review files if the reviewer signed the comments to authors, or if reviewers explicitly agree to release their name. For more information, please refer to our FAQ page.

ORCID
IMPORTANT: Non-corresponding authors do not have to link their ORCIDs but are encouraged to do so. Please note that it will not be possible to add/modify ORCIDs at proof. Thus, please let your co-authors know that if they wish to have their ORCID added to the paper they must follow the procedure described in the following link prior to acceptance:
https://www.springernature.com/gp/researchers/orcid/orcid-for-nature-research

Author names using non-Roman characters
Nature Portfolio journals can support presentation of author names using non-Roman characters in the HTML version of the article. If you wish to, please include author names in parentheses after the Roman-character spelling; see example online here. Currently supported scripts are: Arabic, Chinese, Cyrillic, Devanagari, Greek, Hebrew, Hangul, Japanese and Persian. You will be asked to verify the rendering is correct at proof stage.

Thank you again for your interest in Nature Methods. Please do not hesitate to contact me if you have any questions. We will be in touch again soon.

Best regards,
Nina

Nina Vogt, PhD
Senior Editor
Nature Methods

Reviewer #1 (Remarks to the Author):

I accept the authors' responses and recommend the manuscript for publication.

I would nevertheless like to make one general remark concerning cytoarchitectonic reference atlases. Even when described as "observer-independent" and published in peer-reviewed high-profile journals, such atlases are rarely subjected to broad collegial expert evaluation within the specialized cellular neuroanatomy community. In practice, their widespread citation does not necessarily reflect consensus endorsement, but rather their availability, integration into major infrastructures, and practical convenience for the neuroimaging community, which lacks however expertise to assess cyto-architecture and accepts atlases for granted.

In this context, the Jülich atlas has achieved high visibility and adoption, particularly through its integration within EBRAINS and related platforms. This visibility, however, should not be conflated with universal acceptance as a consensus cytoarchitectonic standard. The cellular neuroanatomy community remains fragmented, and no single parcellation—including Jülich—has achieved full disciplinary consensus.

This does not invalidate the present manuscrip and SUBRA is truly an achievement a great service to the community, but it is an important contextual point when discussing the status and authority of reference atlases within multimodal neuroinformatics frameworks.

Version 2:

Decision Letter:

15th Jun 2026

Dear Timo,

I am pleased to inform you that your Article, "Siibra: A software tool suite for realizing a Multilevel Human Brain Atlas from complex data resources", has now been accepted for publication in Nature Methods. The received and accepted dates will be March 18th, 2025 and June 15th, 2026. This note is intended to let you know what to expect from us over the next month or so,

and to let you know where to address any further questions.

Over the next few weeks, your paper will be copyedited to ensure that it conforms to Nature Methods style. Once your paper is typeset, you will receive an email with a link to choose the appropriate publishing options for your paper and our Author Services team will be in touch regarding any additional information that may be required. It is extremely important that you let us know now whether you will be difficult to contact over the next month. If this is the case, we ask that you send us the contact information (email, phone and fax) of someone who will be able to check the proofs and deal with any last-minute problems.

After the grant of rights is completed, you will receive a link to your electronic proof via email with a request to make any corrections within 48 hours. If, when you receive your proof, you cannot meet this deadline, please inform us at rjsproduction@springernature.com immediately.

Authors may need to take specific actions to achieve compliance with funder and institutional open access mandates. If your research is supported by a funder that requires immediate open access (e.g. according to Plan S principles or the NIH public access policy) then you should select the gold OA route, and we will direct you to the compliant route where possible. Because authors warrant under our subscription licensing terms that they haven’t committed to licensing any version of their article under a licence inconsistent with the terms of our agreement – including the applicable embargo period – publication under the subscription model isn’t suitable for authors whose funders require no embargo.

If you have any questions about our publishing options, costs, Open Access requirements, or our legal forms, please contact ASJournals@springernature.com

If you have posted a preprint on any preprint server, please ensure that the preprint details are updated with a publication reference, including the DOI and a URL to the published version of the article on the journal website.

You may wish to make your media relations office aware of your accepted publication, in case they consider it appropriate to organize some internal or external publicity. Once your paper has been scheduled you will receive an email confirming the publication details. This is normally 3-4 working days in advance of publication. If you need additional notice of the date and time of publication, please let the production team know when you receive the proof of your article to ensure there is sufficient time to coordinate. Further information on our embargo policies can be found here: https://www.nature.com/authors/policies/embargo.html

To assist our authors in disseminating their research to the broader community, our SharedIt initiative provides you with a unique shareable link that will allow anyone (with or without a subscription) to read the published article. Recipients of the link with a subscription will also be able to download and print the PDF.

As soon as your article is published, you will receive an automated email with your shareable link.

You can now use a single sign-on for all your accounts, view the status of all your manuscript submissions and reviews, access usage statistics for your published articles and download a record of your refereeing activity for the Nature journals.

Please note that you and any of your coauthors will be able to order reprints and single copies of the issue containing your article through Nature Portfolio's reprint website, which is located at http://www.nature.com/reprints/author-reprints.html. If there are any questions about reprints please send an email to author-reprints@nature.com and someone will assist you.

Please feel free to contact me if you have questions about any of these points.

Best regards,
Nina

Nina Vogt, PhD
Senior Editor
Nature Methods

** Visit the Springer Nature Editorial and Publishing website at www.springernature.com/editorial-and-publishing-jobs for more information about our career opportunities. If you have any questions please click here.**

Open Access This Peer Review File is licensed under a Creative Commons Attribution 4.0 International License, which permits use, sharing, adaptation, distribution and reproduction in any medium or format, as long as you give appropriate credit to the original author(s) and the source, provide a link to the Creative Commons license, and indicate if changes were made. In cases where reviewers are anonymous, credit should be given to 'Anonymous Referee' and the source.
The images or other third party material in this Peer Review File are included in the article's Creative Commons license, unless indicated otherwise in a credit line to the material. If material is not included in the article's Creative Commons license and your intended use is not permitted by statutory regulation or exceeds the permitted use, you will need to obtain permission directly from the copyright holder.
To view a copy of this license, visit https://creativecommons.org/licenses/by/4.0/

Point-to-point responses to reviewer comments

Our responses are formatted in green. Citations of modified manuscript parts are indented and in italics; modified content formatted in blue. We also included screenshots of new figures inline for convenience.

Reviewer #1

General comment

Dickscheid et al. present "siibra" (Software Interfaces for Interacting with BRain Atlases), a comprehensive open science software suite designed to support the development of a multimodal human brain atlas by integrating diverse multiscale data resources into a coherent anatomical template. It offers a web viewer, a Python library, and an HTTP API, aiming to facilitate access from macroscopic structures down to cellular details. The authors accurately identify and address key challenges involved in multimodal data integration, including interoperability, reproducibility, and scalability. Siibra effectively leverages cloud resources from the EBRAINS infrastructure and other repositories, providing efficient data retrieval and visualization for interactive exploration, computational analysis, and application development. The manuscript systematically guides readers through the extensive features of siibra, which is indeed necessary given the complexity of the data and potential applications. Despite the impressive work underlying siibra and its fantastic potential utility, my hands-on experience revealed significant practical limitations.

We thank the reviewer for the positive feedback regarding the relevance, structure and methodology of the paper, and for acknowledging the challenges of multimodal data integration which the tool suite addresses. We regret that some practical issues were encountered with the software. We respond to each of these points individually in the following.

Specific comment #1: Choice and acceptance of Julich-Brain atlas

1. The manuscript claims that siibra “combines complementary reference atlases reflecting different principles of brain organization.” However, I was expecting to find additional well-established cytoarchitectonic atlases, such as those by Paxinos and Mai. Instead, siibra users currently explore neuroanatomy primarily through the Jülich atlas, which by no means represents a consensus standard. This atlas predominantly covers the cerebral cortex and a limited number of subcortical regions. Thus, it cannot be considered a comprehensive neuroanatomical reference. Moreover, for most templates, the parcellation appears incomplete, which limits the software’s practical utility. A more comprehensive discussion of this aspect is needed.

The results in the manuscript refer to siibra’s default configuration, which uses Julich-Brain and BigBrain as reference contents, reflecting cytoarchitecture as a gold standard. They were chosen because they provide a unique multi-scale approach from the micro- to the macroscale: Julich-Brain is the most detailed cortical atlas, going beyond the historical map of Brodmann as well as the more recent Atlas of the Human Brain by Mai, Majtanik and Paxinos: Cortical areas have here been defined based on observer independent mapping in ten human postmortem brains, thus being unique in capturing inter-individual variability and providing reproducibility based on clearly defined objective mapping criteria (cf. Amunts et al.; Science 2020). The maps available in the atlas have been published in scientific papers, with detailed peer-reviewed information on every single area. This rigorous mapping approach

is linked to a disadvantage - it took more than 20 years to develop the atlas, and some remaining uncharted areas are subsumed under "gap maps".

Regarding the comment of an incomplete parcellation on the side of the reviewer, we want to stress the point that new maps are continuously released to eliminate the remaining gaps. In particular, the upcoming version 3.2 of Julich-Brain includes 162 cortical areas (compared to 43 in Brodmann's and 150 in von Economo's map) and 100 subcortical structures mapped in both hemispheres, totalling 524 maps for both hemispheres in the so-call high granularity version. The remaining GapMaps consist of an limited number of areas where mapping is still in progress. Their size is decreasing with each release of Julich-Brain (v2.9: 38.2%, v3.0.3: 36.7%, v3.1: 25.4%, v3.2: 18%). While many of these maps have been delineated in the BigBrain as one of the ten analysed brains, the BigBrain contains in addition high-resolution 3D maps from an AI-supported workflow (Schiffer et al., 2021) with continuous release cycles. The current set includes 122 maps (61 regions mapped in both hemispheres). The upcoming version will provide more than 50 additional maps and be released in the first half of 2026.

New reference parcellations can be easily integrated into the toolsuite. As described in the section "Separation of content from code", siibra is specifically designed for extension with new content. The default configuration already includes various complementary maps, e.g. white matter architecture (Guevara et al., 2012) and function (Dadi et al., 2020). Following the reviewer's remarks, we further added the von Economo parcellation map in MNI space and provide a snapshot overview of presently available parcellations in the new supplementary table S4. We also examined the possibility of integrating the Mai/Paxinos atlas. Unfortunately, there is no public repository where data are openly available, so we could not include it under the FAIR principles. To make the maps openly available is, however, a key principle of our atlas ecosystem. From a technical point of view, it is straightforward to integrate those maps and others.

We have modified the corresponding paragraph in the main section (p. 3) to better reflect the above points:

> *A central element of the Multilevel Human Brain Atlas is the Julich-Brain cytoarchitectonic atlas [5], which includes probabilistic maps of 262 cortical and subcortical areas for each hemisphere to date. The Julich-Brain atlas was chosen due to its rigorous multi-scale approach. (i) it captures regional microstructural variance based on studies of ten post-mortem brains; (ii) it provides reproducible, observer-independent mapping; (iii) the quality of the mapping has been validated through numerous peer-reviewed publications for all areas; and (iv) it includes a microscopic template space, the BigBrain, enabling to bridge the macro scale (as represented in the MNI template) with the micro scale (as represented in BigBrain). An increasing number of the mapped areas are annotated as detailed 3D maps across the entire series of BigBrain histological sections with 20 µm isotropic resolution [63]. The same methodology used for the ten postmortem brains was used to map the areas in the BigBrain, supplemented by a deep learning workflow. Deep learning was instrumental in speeding up mapping in intermediate sections to achieve dense mapping of areas over their full extent [63]. At present, due to the incremental mapping approach of Julich-Brain, the probabilistic maps include some uncharted areas, which are subsumed under 'GapMaps', and the set of full-resolution BigBrain maps is not yet complete-it consists of 122 maps (61 per hemisphere) to date. An overview of the parcellations included in the current version of the Multilevel Human Brain Atlas can be found in Fig. S4. In line with our vision of a 'living atlas', updates with new areas are being released at a rate of 1–2 per year (see section 'Expansion with new content'). Such thorough mapping remains very time-consuming, with an estimated effort of 1 area per year and scientist.*

We have also examined the usage of that atlas in more detail: Julich-Brain and BigBrain are becoming well adopted systems, as evidenced by their growing number of citations. The original paper introducing

the BigBrain (Amunts et al., Science 2013, DOI: 10.1126/science.1235381) has by now received 1059 citations, and the original paper of Julich-Brain has received more than 588 citations (Amunts at al., Science 2020, DOI: 10.1126/science.abb4588). According to Google Scholar, about 10,000 citations have been made regarding the original publications of brain areas including the SPM toolbox. Moreover, we observe steadily growing numbers of visitors in our online services at EBRAINS, with 3000 - 4000 unique visitors per month of the online viewer siibra-explorer and a similar number of accesses to the underlying datasets for Julich-Brain in the EBRAINS knowledge graph. Acceptance of Julich-Brain in the community is also underpinned by providers of popular neuroimaging packages, who include or reference recent versions of Julich-Brain in their distributions. Concrete examples are

- AFNI (v3.0; https://afni.nimh.nih.gov/pub/dist/doc/program_help/whereami.html)
- FSL (https://fsl.fmrib.ox.ac.uk/fsl/docs/#/other/datasets?id=julich-brain-cytoarchitectonic-atlas-atlas)
- niilearn (https://nilearn.github.io/dev/modules/description/juelich.html)
- LeadDBS (https://www.lead-dbs.org/helpsupport/knowledge-base/atlasesresources/cortical-atlas-parcellations-mni-space/)

Thus, we would argue that Julich-Brain and Bigbrain are well known, and that they are used as a standard atlas by a large and constantly growing community of researchers.

References:

P. Guevara, D. Duclap, C. Poupon, L. Marrakchi-Kacem, P. Fillard, D. Le Bihan, M. Leboyer, J. Houenou, and J. F. Mangin. Automatic fiber bundle segmentation in massive tractography datasets using a multi-subject bundle atlas. NeuroImage, 61(4):1083–1099, July 2012. ISSN 1053-8119. doi: 10.1016/j.neuroimage.2012.02.071.

K. Dadi, G. Varoquaux, A. Machlouzarides-Shalit, K. J. Gorgolewski, D. Wassermann, B. Thirion, and A. Mensch. Fine-grain atlases of functional modes for fMRI analysis. NeuroImage, 221:117126, November 2020. ISSN 1053-8119. doi: 10.1016/j.neuroimage.2020.
117126.

Parcellation	Space	Map	Ref.	Panel
Julich-Brain	ICBM 2009c asym	Cytoarchitectonic probabilistic maps	[5]	A
	MNI Colin 27	Cytoarchitectonic probabilistic maps	[5]	B
	BigBrain	High-resolution cytoarchitectonic 3D maps	[?]	G
	fsaverage	Cytoarchitectonic maximum probability map	[5]	I
Fibre Bundles	ICBM 2009c asym	Deep white matter bundle maps	[35]	D
	ICBM 2009c asym	Superficial bundle maps	[35]	E
Von Economo	ICBM 2009c asym	Cytoarchitectonic map	[72]	C
DiFuMo	ICBM 2009c asym	Map of 64 functional modes	[20]	
	ICBM 2009c asym	Map of 128 functional modes	[20]	
	ICBM 2009c asym	Map of 256 functional modes	[20]	
	ICBM 2009c asym	Map of 512 functional modes	[20]	
	ICBM 2009c asym	Map of 1024 functional modes	[20]	F
Cortical layers	BigBrain	Atlas of cortical layers	[?]	H

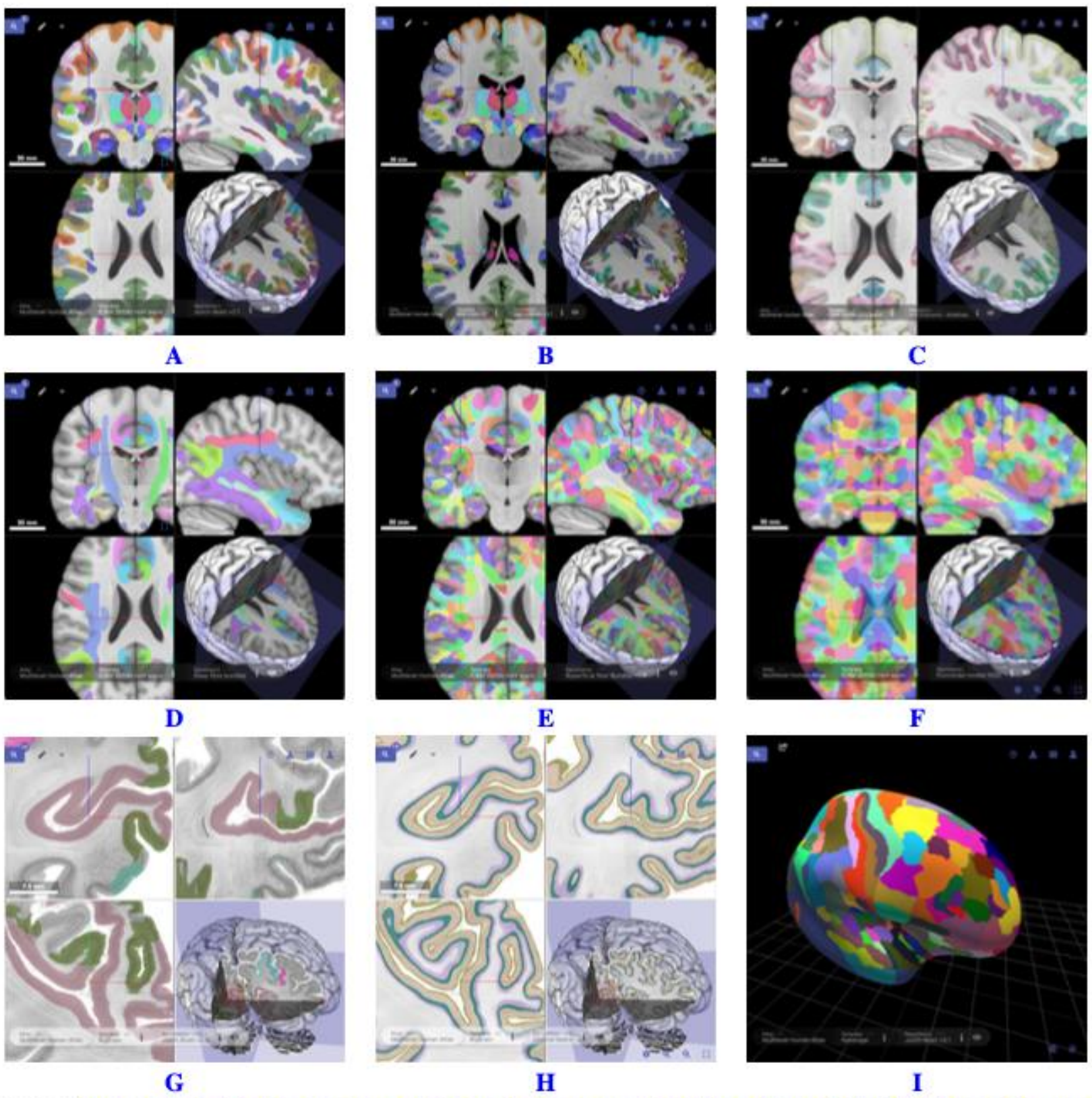

Table S4 | **Snapshot of reference parcellations in the current configuration of the Multilevel Human Brain Atlas.** The table summarizes the set of parcellation maps included in default *siibra* configuration of the Multilevel Human Brain Atlas at the time of writing. The reported version of the configuration is available at `https://github.com/FZJ-INM1-BDA/siibra-configurations/tree/v1`. The system is designed to be extended with more parcellations and versions. Local installations of *siibra* further allow to use configurations with custom maps.

Specific comment #2: Switching reference spaces

2. Testing a specific workflow, I selected area 4 (primary motor cortex) and attempted to switch from MNI Colin27 (JulichBrain 2.9) to the BigBrain template (same atlas). Contrary to the authors' statements, siibra did not maintain the anatomical location. Instead of remaining focused on area 4, the viewer unexpectedly shifted to area 6d. Using the cortical layer atlas of BigBrain resulted in better anatomical alignment, but the zoom level was lost during the transition. Thus, the described "precomputed nonlinear spatial transformations" did not effectively preserve location and zoom levels.

We thank the reviewer for testing the workflow in Fig. 2. We have reproduced the issue and believe that the impression of an incorrect coordinate mapping arises from a misunderstanding, which we addressed with a software update. We will explain the behaviour step by step and provide URLs for reproduction:

1. The workflow starts from motor area 4 in MNI colin space. We have here chosen 4p in the right hemisphere:
   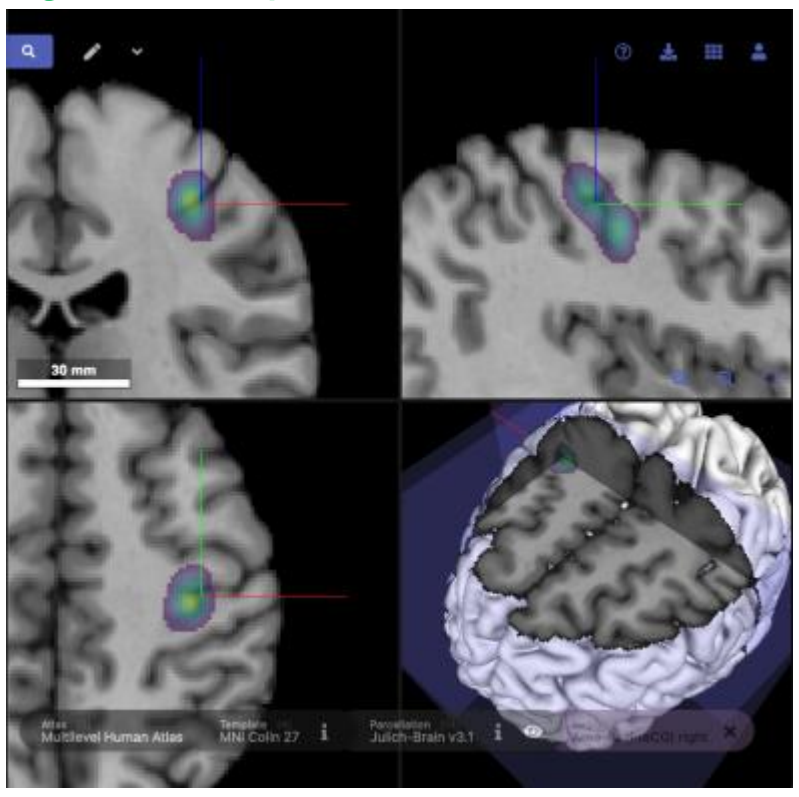
   https://atlases.ebrains.eu/viewer/#/a:juelich:iav:atlas:v1.0.0:1/t:minds:core:referencespace:v1.0.0:7f39f7be-445b-47c0-9791-e971c0b6d992/p:minds:core:parcellationatlas:v1.0.0:94c1125b-b87e-45e4-901c-00daee7f2579-310/rn:9184c35/@:0.0.0.-W000.._hVJB.2-PtJd._Uc6Q.2zhrgC..8iGa..2MF44.1J3F4~.2we74.._38/vs:v2-ff011b03
2. From here the reviewer switched to the BigBrain space. The implemented action is that position and zoom level (in terms of mm per pixel dimension) are preserved, while the previous area selection is ignored. Thus, while the target view navigates to the corresponding location and preserves orientation and zoom level, it displays all available maps instead of the previously selected one. In this specific case the crosshair is positioned in an uncharted region, because the high-resolution map of area 4p in BigBrain space is not yet released. The closest visible map is indeed that of area 6d1 (in dark gray in the axial plane), which may have caused the confusion experienced by the reviewer.
   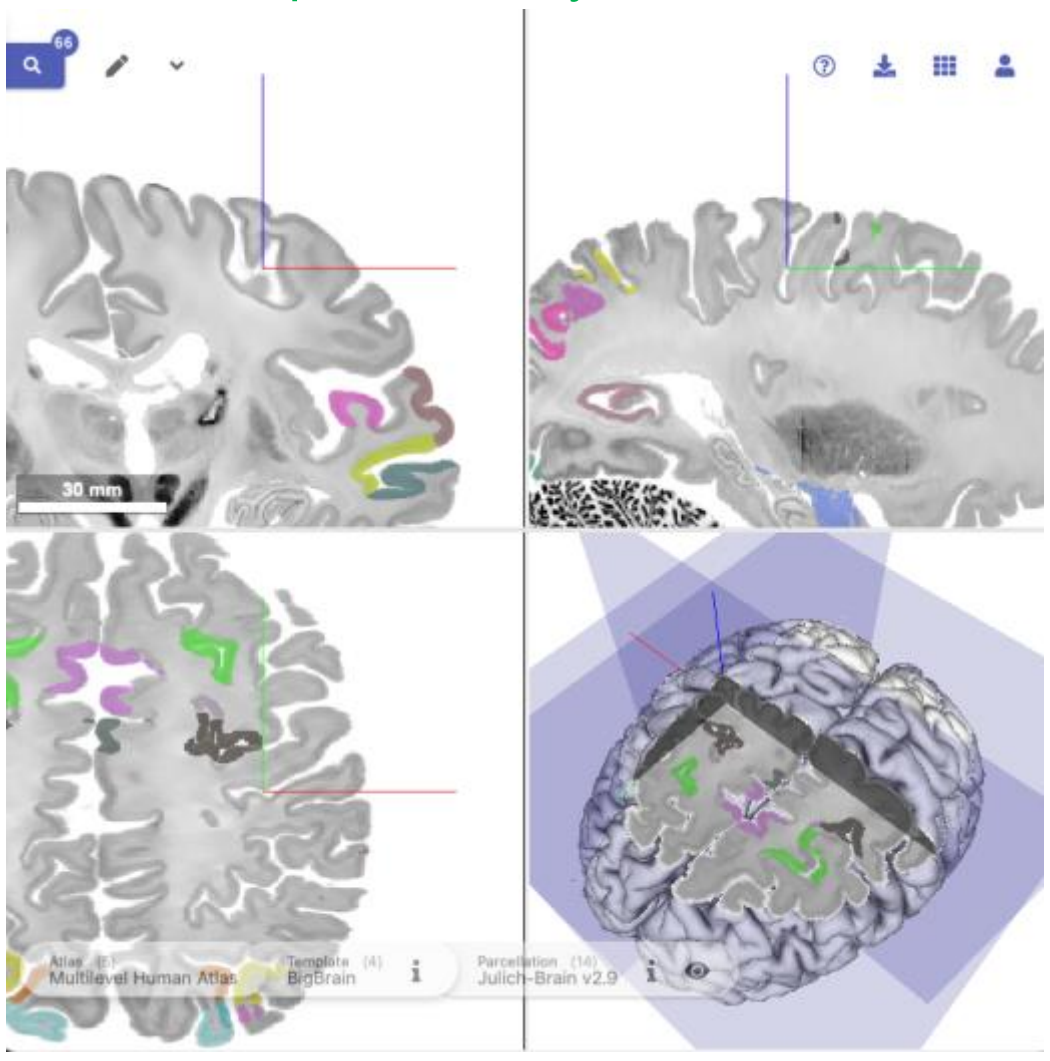
   https://atlases.ebrains.eu/viewer/#/a:juelich:iav:atlas:v1.0.0:1/t:minds:core:referencespace:v1.0.0:a1655b99-82f1-420f-a3c2-fe80fd4c8588/p:minds:core:parcellationatlas:v1.0.0:94c1125b-b87e-45e4-901c-00daee7f2579-290/@:0.0.0.-W000.._hVJB.2-PtJd._Uc6Q.2zhrgC..8iGa..1To_e.BzF0.213iK.._38/vs:v2-ff011b03

To improve the user experience and remove ambiguities, we modified the functionality as follows: Siibra-explorer will now preserve a selected region when switching the reference space, in so far, a map is available in the target space. If this is not the case, as t was the case in the present example, the region selection will be cleared, but a message stating as such will be displayed to inform the user. In the present case, the message reads:

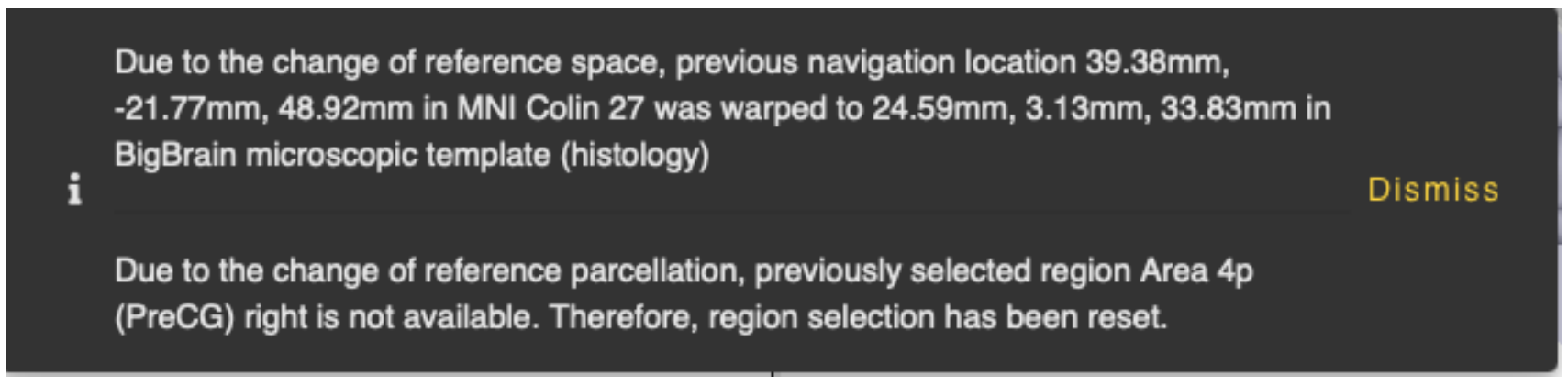

We revised the description of the template switching mechanism accordingly and extended the description in the results section (page 5):

> *When switching to the BigBrain template, precomputed nonlinear spatial transformations are used to preserve location while changing the reference coordinate system (Figure 2C). Besides inevitable trade-offs required in nonlinear cross-subject registration, as described e.g. in [68], the precision of this coordinate translation depends on numerical limitations and the degree of nonlinear deformation between the templates in the zoomed-in region of interest. A more detailed assessment of the expected precision is given in Sec. "Modeling spatial entities in different reference coordinate systems" and supplementary Figure S5.*
> *If a particular brain area is selected in the source reference space, such as in Figure 2B, siibra-explorer tries to preserve the selection in the target space. However, a corresponding map of the region might not be available in the target space. In this case, the area selection is discarded with a corresponding notification, and the corresponding region of interest is displayed as uncharted in the target reference space."*

In the methods section (page 15), we further included an analysis of the expected precision when preserving locations across coordinate systems:

> *"[...] The precision of cross-subject matching using these spatial transformations has been evaluated for cortex overlap and sulcal dispersion in [46]. […] To provide an additional assessment of their practical application for coordinate warping in siibra, we carried out two experiments, with results compiled in supplementary Figure S5:*
>
> 1. *Reprojection errors were computed when warping random gray matter positions from one template space to another and back (Figure S5A). The experiment was performed with N=5000 points between the ICBM 2009c nonl asym, Colin 27 and BigBrain spaces. Average reprojection errors were in the range of 10-20 µm, occasionally reaching the range of 100-200µm. These are attributed to numerical limitations arising from the large differences between the spaces and the need for serialization of the floating numbers during network communication.*
> 2. *For cytoarchitectonic brain areas mapped in BigBrain, 500 random locations each were sampled uniformly from the ultrahigh-resolution 3D BigBrain map, and warped to the ICBM 2009c nonl asym space. Their probability to lie consistently inside the same brain area was then determined in the target space using the corresponding Julich-Brain probabilistic map (Figure S5C). Except for occasional outliers close to areal borders, warped points were located at nonzero probabilities for all evaluated brain areas, while the average probability score varied significantly between the regions. This can be expected, because the probability maps in MNI space capture the variance of mappings from ten different postmortem brains [5] after spatial normalization, and the individual subject map in BigBrain space does not necessarily coincide with their average."*

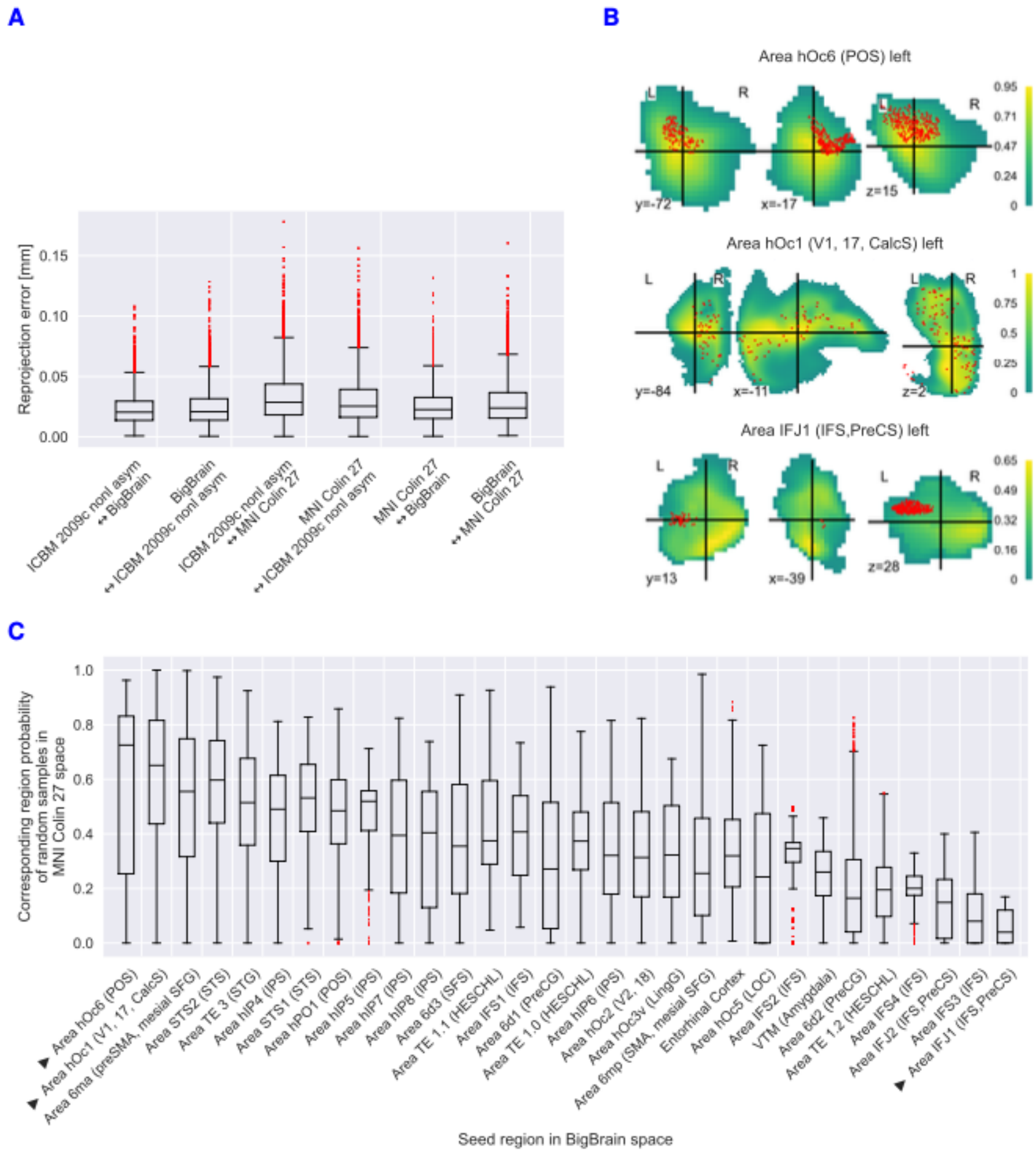

Table S5 | **Quantitative assessment of coordinate warping applied in *siibra*.** **A**: Distribution of reprojection errors (in mm) after warping $N = 5000$ randomly sampled gray matter locations from a source to a target reference space. While the underlying nonlinear transformations [48] are diffeomorphisms, numerical inaccuracies in the order 10-20 micrometer have to be expected, occassionally reaching up to about 100 micrometer. **B, C**: Warping of $N = 500$ random coordinates that were sampled uniformly from each ultrahigh-resolution cytoarchitectonic map in BigBrain space [65] to MNI ICBM 2009s asymmetric space. **C**: Probabilities obtained by re-assigning the warped coordinates to the same brain region using Julich-Brain probabilistic maps [5] in MNI space. **B**: Plot of warped random samples for three example regions (marked by "▶" in C) on top the respective probability maps to illustrate the variation.

Specific comment #3: Performance and scalability benchmarks

Quantitative benchmarks demonstrating performance and scalability would greatly reinforce claims about siibra's ability to manage large-scale data.

We agree with the reviewer that an assessment of the performance and scalability of siibra is important, in particular when using large data. Therefore, we measured response times for a range of workflows, including the one illustrated in Fig. 1 of the manuscript, from different physical locations in Europe, North America, Asia, and Australia. These have been added as new supplementary Fig. S6. We observed that a smooth performance of the system can currently be expected in Europe, while the experience in other continents is not always satisfactory. Overcoming this problem requires to develop other technical solutions, e.g. to deploy mirrors of siibra's contents at different physical locations around the world. Smooth exchange of data for research purposes can be supported by certain organisation, but we feel that this kind of question goes beyond the scope of the siibra toolbox. Since siibra currently relies on availability of data on existing cloud repositories, mostly EBRAINS, this will also require consultation and cooperation with those service providers. We have added a brief paragraph addressing this limitation to the discussion section of the manuscript:

Discussion, page 12:

"[...] The practical performance of accessing large datasets with siibra depends on the network connection between the user's client machine (e.g. the web browser running siibra-explorer) and the respective data service hosting the requested content. At present, most of siibra's contents are hosted on EBRAINS, which uses storage systems on European supercomputing centers. As a result, the performance of siibra is more fluid and reliable inside Europe than from other continents including North America, Asia and Australia. Figure S6 includes an analysis of response times when running the workflow in Figure 2 repeatedly from different geolocations, which quantifies these differences. Setting up a globally distributed ecosystem of cloud resources to provide a well-balanced performance from different geographic locations is a common challenge for big data services, and part of our future considerations to extend the framework."

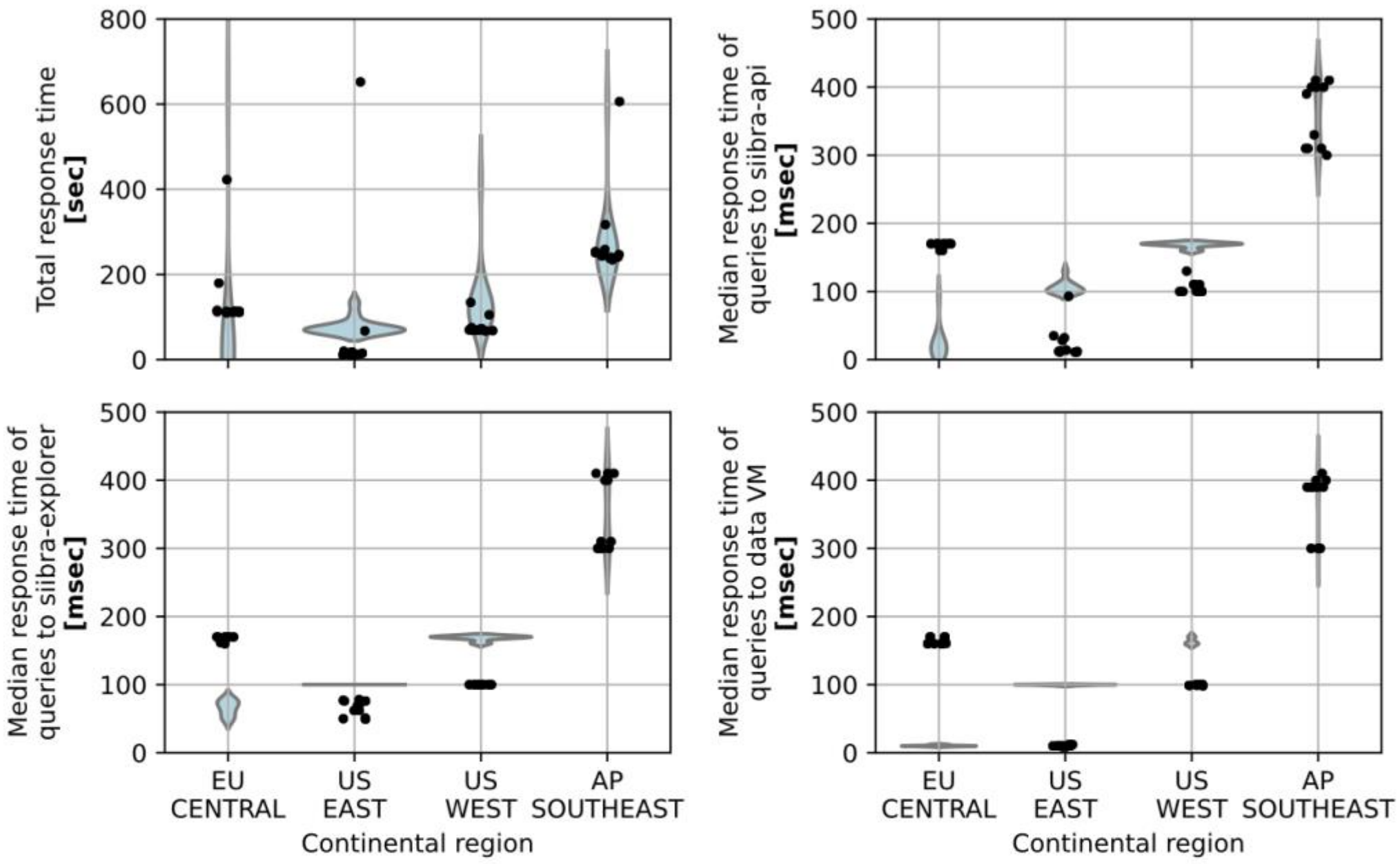

Figure S6 | Response times of a typical access pattern from different continents. The boxplots summarize the distribution of response times measured when performing the scenario in Figure 2 in *siibra* repeatedly from different continents. Measurements were carried out by recording the HTTP requests of the workflow as an Ansible playbook, and running the same requests from virtual machines (1 CPU, 1GB RAM, 40 Gbps / 1 Gbps network in/out) provisioned on Linode located on pre-specified geographical locations. Playbooks were executed at least ten times on different dates during August and September 2025. We report the total response time in milliseconds of each playbook run. From top left to bottom right: Total response times, response times to the subset of requests to *siibra-api*, *siibra-explorer* and underlying virtual machines serving data.

Specific comment #4: Navigation when selecting new regions

3. When exploring the precentral gyrus, no anatomical area was interactively highlighted. The structural list further indicated that Areas 4a and 4p were "not mapped in this template space," raising concerns about completeness and consistency of mappings within siibra. Additionally, a dedicated section explicitly discussing limitations or potential barriers to widespread adoption, such as network dependency, hardware requirements, or integration of novel data types, is notably missing and would significantly enhance the manuscript.

The reviewer correctly noticed an uncharted brain region in one of the template spaces, the high-resolution BigBrain template, while the same areas are available as probability maps in MNI space. As discussed above (Specific comment #2), we modified the software to offer a more consistent behaviour and clear notifications when navigating to uncharted brain regions. We are working on synchronizing

the number of mapped regions in both the BigBrain and the MNI templates in the near future. We also agree that long response times, mostly experienced outside Europe, may result in unsatisfactory user experience, and have added a quantitative assessment and explanation in the discussion, as described for the previous remark (Specific comment #3).

Specific comment #5: Layer 4 in agranular regions

4. I also question the anatomical validity of representing layer 4 in the agranular primary motor cortex. Maybe the authors referred to a paper by Helen Barbas on the apparent quasi acellular gap between layers 3 and 5, which is likely where many thalamocortical projections terminate.

We appreciate the reviewer's comment. Layer IV in agranular regions is not present in the adult human cortex, but only visible during early postnatal ontogeny. Later it disappears as a distinct layer around months 8 (Marin-Padilla, 1970, Amunts et al., 1995). The cortical layer segmentation approach published by Wagstyl et al. (2019) does not distinguish between granular and agranular areas. Their model was trained on a limited number of manually annotated regions and does not explicitly account for regional deviations from the canonical six-layered isocortical structure. The siibra toolsuite allows to integrate this model, but does not compute the layers. This holds for any content in siibra, as described in section "Separation of content from code" (p. 16 of the manuscript). A concise description of the methodology and direct link to the open-access publication are accessible via the information ("i") button in siibra-explorer, and can also be accessed through object methods in siibra-python. We added the following disclaimer to the existing information text in the siibra-explorer:

> *"Regional inaccuracies in the automated computation of cortical layers may occur due to limitations in the available training data and algorithm. Regions that deviate from the expected canonical isocortical structure should be examined with caution."*

Reference:

Wagstyl, K., et al. (2019). *BigBrain 3D atlas of cortical layers: Cortical and laminar thickness gradients diverge in sensory and motor cortices.* PLOS Biology. https://doi.org/10.1371/journal.pbio.3000289

Marin-Padilla, Miguel (1970). *Prenatal and Early Postnatal Ontogenesis of the Human Motor Cortex: A Golgi Study. I. The Sequential Development of the Cortical Layers*. Brain Research 23 (2): 167–83. https://doi.org/10.1016/0006-8993(70)90037-5.

Amunts, Katrin, Vadim Istomin, Axel Schleicher, and Karl Zilles (1995) *Postnatal Development of the Human Primary Motor Cortex: A Quantitative Cytoarchitectonic Analysis*. Anatomy and Embryology 192 (6): 557–71. https://doi.org/10.1007/BF00187186.

Specific comment #6: too high latency for high-resolution data

5. In attempting to reproduce Figure 2's high-resolution cellular detail, after more than 10 minutes, the displayed image remained pixelated, and the promised cellular resolution was not achievable in practice.

We regret the disappointing experience with siibra-explorer when the reviewer tried to reproduce Fig. 2. We performed the same workflow many times and observed fluid performance. The most likely explanation is that no 1 micrometer scan was selected from the spatial search results in step C of Fig. 2, which is necessary to reveal the high level of detail shown in Fig. 2 D-E. Indeed, thanks to the reviewer's remark, we realized that Fig. 2 should be more explicit about this step. We thus modified the image in Fig. 2C to show the spatial search explicitly, and updated its caption accordingly:

> “[..] When switching to the microscopic BigBrain model as a reference space [4], the level of detail is significantly increased while siibra-explorer preserves zoom and location. The parcellation is changed to cortical layer maps [70]. Additional parcellations and anchored image data are revealed. A 1µm scan of a coronal BigBrain section is selected from the spatial search results to reveal full resolution. [..]”

As described above, we further verified in a new experiment that the performance depends significantly on the geographic location and can be unsatisfactory in countries outside Europe. The results of this analysis were added as a new Fig. S6, and a critical reflection was incorporated in the discussion section. However, we do not assume that performance problems were reason for the issue encountered here, since we could not observe delays in the order of multiple minutes.

Specific comment #7: multimodal comparison

6. During my second test (Multimodal comparison of brain areas), selecting area 44 via the search menu (MNI Colin 27 + JB v3.1) inexplicably navigated to area 3b instead. Given my limited experience with Python, I did not test data retrieval via scripts. However, if this retrieval mechanism functions as demonstrated in Figure 4, it would indeed represent a powerful and valuable feature of siibra.

We thank the reviewer for pointing us to this rather unintuitive behaviour. Indeed, in the software version that was published when we submitted the manuscript, siibra-explorer did not automatically navigate to the new position after selecting a brain region from the region search - it modified the selection but preserved the viewing position. We have modified the behaviour accordingly in the updated version of siibra-explorer. Selection of area 44 will now automatically navigate to the centroid of that region and thus avoid this problem.

In conclusion, while siibra represents an ambitious and theoretically powerful solution for neuroanatomical exploration, these significant practical issues, along with gaps in benchmarking, comparative analysis, and explicit discussion of limitations, currently limit its utility and accessibility. Addressing these concerns would substantially enhance the effectiveness, reliability, and clarity of this promising tool.

We thank the reviewer for the precise analysis and the accurate assessment of the various points in our manuscript, which have helped us to improve the manuscript. We hope that we could adequately address these with the mentioned software updates and newly incorporated analyses and explanations in the revised text.

Reviewer #2

Summary of Key Results: The manuscript presents Siibra, a multiscale neuroinformatics platform designed to integrate diverse human brain datasets into a harmonized atlas framework. It supports multimodal analyses by enabling spatial alignment of molecular, anatomical, and functional data across reference templates, with open-access implementation and adherence to FAIR principles.

Originality and Significance: The work is technically original and addresses a critical infrastructure gap in multilevel brain data integration. While atlas-based frameworks exist, Siibra’s emphasis on interoperability, extensibility, and integration of high-resolution modalities (e.g., BigBrain histology) makes it a meaningful advance in open neuroinformatics.

We thank the reviewer for this positive assessment of our work.

Data and Methodology: The platform leverages well-structured metadata standards (e.g., openMINDS), modular architecture, and cloud-based delivery mechanisms. While these aspects are well-documented, the manuscript would benefit from a clearer validation framework to assess anatomical congruency and transformation fidelity. The lack of a demonstrative analysis case limits the methodological impact.

We are grateful for this important remark. While the quality of spatial transformations has been analysed in the referenced paper by Lebenberg et al., we agree that it is not sufficiently summarized and validated in practice for the application in siibra. We therefore carried out an additional quantitative validation of coordinate warpings, compiled as a new Figure S5, and summarized them in the methods section. Please refer to our detailed remarks to reviewer 1 above (Specific comment #2). The relevant added section in the methods (p. 13/14) is:

> *"[...]The precision of cross-subject matching using these spatial transformations has been evaluated for cortex overlap and sulcal dispersion in [46]. To provide an additional assessment of their practical application for coordinate warping in siibra, we carried out three different experiments, with results compiled in supplementary Figure S5:*
>
> 1. *Reprojection errors were computed when warping random gray matter positions from one template space to another and back (FigureS5A). The experiment was performed with N=5000 points between the ICBM 2009c nonl asym, Colin 27 and BigBrain spaces. Average reprojection errors were in the range of 10-20 µm, occasionally reaching the range of 100-200µm. These are attributed to numerical limitations arising from large scale differences between the spaces and the need for serialization of the floating numbers during network communication.*
> 2. *For cytoarchitectonic brain areas mapped in BigBrain, 500 random locations each were sampled uniformly from the ultrahigh-resolution 3D BigBrain map, and warped to the ICBM 2009c nonl asym space. Their probability to lie consistently inside the same brain area was then determined in the target space using the corresponding Julich-Brain probabilistic map (Figure S5C). Except for occasional outliers close to areal borders, warped points were located at nonzero probabilities for all evaluated brain areas, while the average probability score varied significantly between the regions. This can be expected, because the probability maps in MNI space capture the variance of mappings from ten different postmortem brains [5] after spatial normalization, and the individual subject map in BigBrain space does not necessarily coincide with their average. [...]"*

Statistics and Uncertainties: The manuscript is not heavily reliant on statistical modeling, but performance evaluations (e.g., query latency, system scalability) would fall under this rubric. At present, such benchmarks are not reported, leaving the platform's operational robustness largely qualitative.

Responding to the issues raised by the reviewer, we have added new quantitative evaluations in Figs. S5 and S6. These include comprehensive statistics as box plots, with median, quantiles and visualization of outlier points.

Conclusions: The conclusions are consistent with the platform's described architecture but could be more robust if supported by a fully developed analytic use case demonstrating real-world application and data-driven inference.

We agree that a concrete analytic use case is a valuable addition for the manuscript. We therefore selected a recent study which proposed connectivity-based subcortical maps derived from in-vivo imaging for brain stimulation, and used siibra to re-assess the anatomical characterization of the maps proposed in this study. Siibra provided a reproducible assignment of the maps to cytoarchitectonically defined subcortical structures, confirming some findings of the study while providing additional

localization details and detailed evidence of the assessment at cellular resolution. The use case is described as a new paragraph in the results section (p. 10), supported with a comprehensive new Figure 6:

> ***„Case study: Anatomical evaluation of subcortical maps***
> *To demonstrate the functionalities of siibra, maps of subcortical structures derived from in-vivo imaging were re-analysed to localize them precisely and link them to microstructural structures. The subcortical maps were taken from a recent study [9], which proposed an in-vivo sub-parcellation of the ventral intermediate nucleus (VIM) based on streamlines extracted from diffusion MRI data in human subjects. In short, the authors of [9] extracted the set of streamlines connecting the thalamus with pre-defined cortical target regions, resulting in structural connectivity profiles for each voxel in the thalamus per subject. Using k-means clustering, voxels in the thalamus were then grouped into sub-regions with similar connectivity profiles, resulting in multiple candidate subthalamic maps, here denoted as cluster maps. The study identified cluster maps with significant spatial overlap to the VIM, using Dice scores with histology-based segmentation masks of VIM [29] projected to the indivdual subject spaces, resulting in a selection of only two relevant cluster maps, Precentral and Dentate. We used siibra-python to reassess the selection using probabilistic assignment (cf. Figure 3), and to verify the situation at cellular resolution using microscopy data extraction (cf. Figure 5). Results are summarized in Figure 6 and reveal several interesting aspects:*
>
> 1. *Assignment of cluster maps to Julich-Brain probabilistic maps in MNI space independently confirmed the selection of the Precentral and Dentate clusters in the study in terms of high correlation coefficients (Figure 6A, center).*
> 2. *For the Dentate cluster, association with VIM was further confirmed as the strongest overall correlation among cytoarchitectonic areas in Julich-Brain (Figure 6A, right).*
> 3. *However, for the Precentral cluster, the strongest correlation was found with the ventral posterior lateral nucleus (VPL) rather than the VIM (Figure 6A, left). This indicates that the Precentral cluster may not be connected to a subpart of VIM, but rather to the adjacent VPL.*
> 4. *Intersection of the different MNI maps with relevant histological sections from the BigBrain model [3; 66] allowed to further examine the localisation at cellular resolution (Figure 6B,C), revealing that the Precentral cluster was indeed located within the borders of VPL, while being only adjacent to VIM (Figure 6C, left). Some sections showed a less distinct yet confirmative association where both VPL and VIM overlap with the Precentral cluster (Figure 6C, center).*
> 5. *In contrast, the Dentate cluster was usually located within the borders of VIM in both Julich-Brain and Distal atlas (Figure 6C, right).*
> 6. *The level of detail in the images retrieved with siibra-python allowed to clearly observe the cytoarchitectonic differences defining the boundary of VIM (Figure 6D), enabling a neuroanatomist to create a reference annotation in one of the sections (Figure 6C, left; in green). The reference annotation confirmed overall plausible location of the VPL and VIM contours projected from MNI to BigBrain space.*
>
> *Thus, siibra-python enabled simple and reproducible assignment of the connectivity-based subthalamic maps to cytoarchitectonically defined subcortical structures, confirming some findings of the published study while providing additional, more fine-grained localization details and links to openly accessible reference parcellations. It further provided detailed evidence of the assessment at cellular resolution by fascilitating access to relevant histological image data and automatically handling the different coordinate systems and data types involved in the process.“*

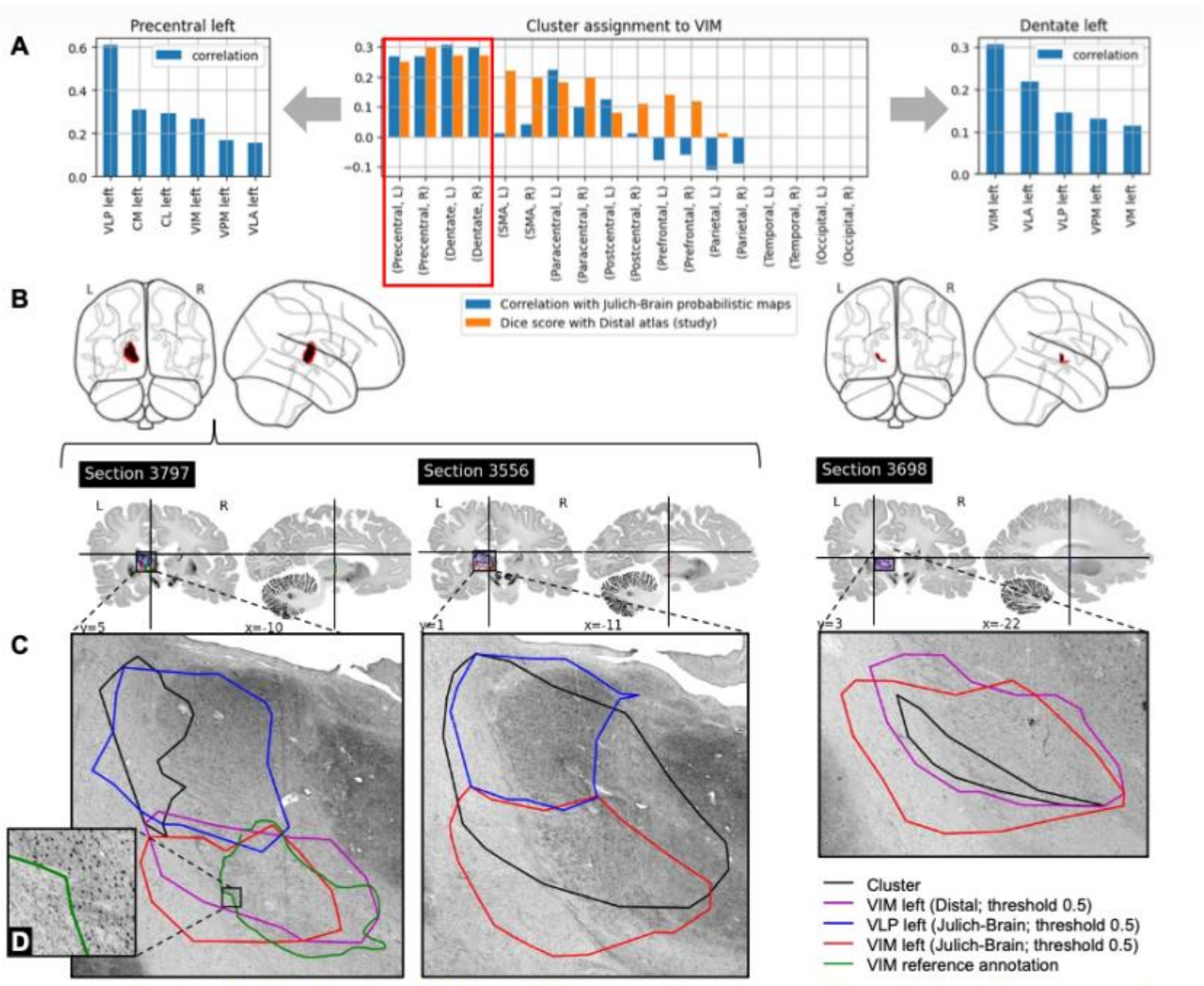

Figure 6 | **Anatomical evaluation of subcortical maps using *siibra-python*.** **A**: Spatial correspondence of connectivity-based subthalamic cluster maps [9] with cytoarchitectonically defined nuclei. Center: Dice scores in individual subject space between cluster maps and the ventral intermediate nucleus (VIM) in the Distal atlas [29] reported in [9], compared to correlation coefficients with the Julich-Brain probabilistic map of VIM [22] computed in MNI152 asymmetric space by *siibra-python* as in Figure 3. Left and right: Correlation coefficients of clusters *Precentral* and *Dentate* (left hemisphere) with other probabilistic maps in Julich-Brain, thresholded at $\rho = 0.25$. **B**: Relevant histological sections from BigBrain [3; 66] identified and retrieved using *siibra-python* based on the Precentral and Dentate cluster maps as in Figure 5. **C**: Assessment of the underlying cytoarchitecture and mapped structures at cellular resolution by intersection of maps with the histological sections. Left: In section 3797, the Precentral cluster (black) lies within the borders of the VPL (blue) in Julich-Brain while being only adjacent to the VIM. Contours of the VIM in Julich-Brain (red) and Distal atlas (magenta) are overall consistent. A reference annotation by a trained neuroanatomist in this section (green) confirms the approximate delineation of VPL and VIM projected from MNI space to BigBrain space. Center: Section 3556, located further posterior, shows a less distinct yet confirmative association where both VPL and VIM overlap with the Precentral cluster. Right: In section 3698, the location of the Dentate cluster (black) lies clearly inside the borders of the VIM in both Julich-Brain (red) and Distal (magenta) as suggested by the overlap scores in A. **D**: Full-resolution view of the retrieved image near the VIM boundary in section 3797, revealing clear cytoarchitectonic differences at the reference delineation of the expert (green). *The Figure can be reproduced using the notebook available at* `https://siibra-python.readthedocs.io/en/v1/examples/tutorials/2025-paper-fig6.html`.

Suggested Improvements:

Include performance metrics illustrating scalability (e.g., latency, rendering load).

We agree that a critical performance analysis will improve the manuscript and help setting realistic expectations for readers. We have measured response times for a range of workflows, including the one illustrated in Fig. 1 of the manuscript, from physical locations on different continents; including countries in Europe, North America, Asia, Africa and Australia. The response times have been added as supplementary Fig. S6. We observed that a fluid performance of the system can currently only be expected in Europe, while the user experience in other continents is often not satisfactory. We added a paragraph addressing this limitation to the discussion section of the manuscript:

Discussion, page 14:

> "*[...] The practical performance of accessing large datasets with siibra depends on the network connection between the user's client machine (e.g. the web browser running siibra-explorer) and the respective data service hosting the requested content. At present, most of siibra's contents are hosted on EBRAINS, which uses storage systems on European supercomputing centers. As a result, the performance of siibra is more fluid and reliable inside Europe, and can be unsatisfactory from inside other continents including North America, Asia and Australia. It will hence be crucial for broad acceptance of siibra as a research tool to setup a globally distributed*

ecosystem of redundant cloud resources and provide well-balanced performance from different geographic locations. Possible strategies need to be explored in suitable infrastructure consortia such as EBRAINS or EuroHPC."

Add a brief but concrete validation strategy across spatial transformations.

As mentioned above, we are grateful for this suggestion and carried out a multifold evaluation of the reliability when warping coordinates across reference space, resulting in the new Figure S5 and additional explanations in the Methods section.

Demonstrate a use case illustrating the tool's analytical value.

As mentioned above, we are grateful for this suggestion and have added a concrete use case to the results section.

Clarify how users can integrate custom datasets into the existing framework.

The integration of new data is described under "Expansion with new contents" in the results section. Integration of custom datasets is specifically mentioned in point 3 (page 11):

"Local content, including both reference atlases and data features, can be added ad-hoc in siibra-python using various utility functions, such as in Figure 5C. For example, users may add a custom parcellation map or an image volume to query data features, demonstrated for the AICHA atlas [39] in the code example available at siibra-python.readthedocs.io. In siibra-explorer, user supplied NIfTI volumes can be visualized on top of existing reference atlases by drag-and-drop onto the browser window, as illustrated in the documentation at siibra-explorer.readthedocs.io. Assuming it has been pre-aligned to the respective reference coordinate space, the file is co-visualized in the browser while solely remaining on the user's local system."

References are generally appropriate and include citations to major resources (e.g., BigBrain, Julich-Brain, EBRAINS). The inclusion of direct comparisons to other atlas-based tools would further contextualize novelty.

Clarity and Context: The manuscript is clearly written, with an informative abstract and appropriate structure. Minor improvements include:

Clarifying units in Supplementary Figure S1 (e.g., "1 pm" likely meant as 1 µm)
Unfortunately, we could not find the string "pm" in the supplementary figures; indeed we assume that 1 µm is the correct meaning.

Specifying default template/parcellation versions in siibra-python
We have added a new Figure S4, which provides an overview of the different parcellation maps included in the current default configuration. We added a reference the figure in the main section on page 2:

"An overview of the reference parcellations in the present version of the Multilevel Human Brain Atlas is provided in Figure S4. However, the system is designed for continuous expansion with additional reference parcellations and future versions of existing ones (Sec. "Expansion with new contents"), appearing side by side without differentiation of their importance."

Including a schematic of metadata relationships to assist new users

Metadata relationships occur on the level of concept in the tool suite, and on the level of entities of the included reference atlases. For the first, we think that they are summarized well in Fig. 7 of the manuscript. We have added this Figure to the documentation of siibra-python. For the latter, users can access the hierarchical region tree for each selected atlas, as illustrated below.

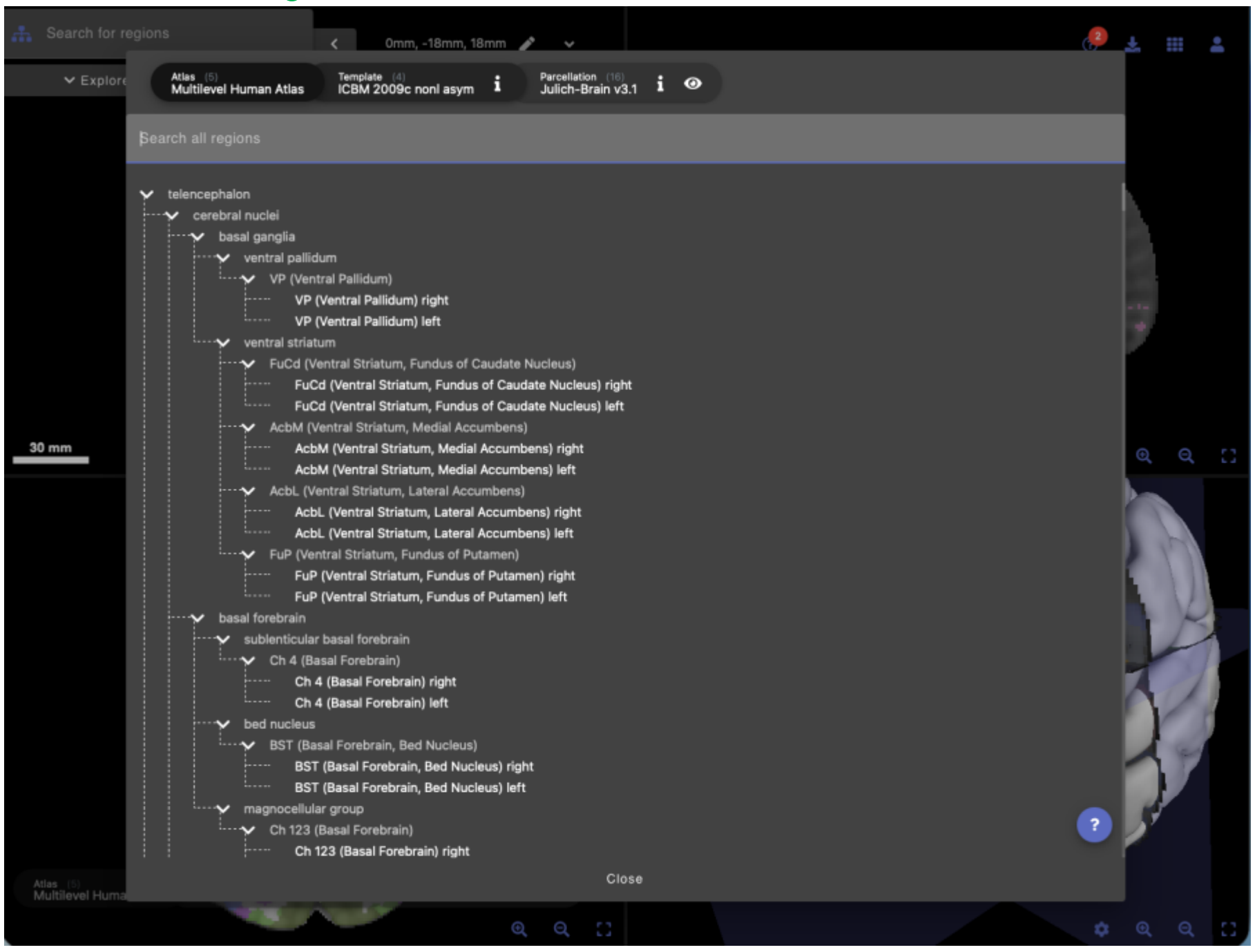

Remarks on code availability:

Each of the three repositories—siibra-python, siibra-explorer, and siibra-api—includes a comprehensive README file with installation instructions, usage examples, and links to further documentation.

siibra-python is pip-installable and includes tested examples via ReadTheDocs. It supports reproducible workflows through versioned dependencies and continuous integration across Python 3.7–3.12 environments.

siibra-explorer provides a browser-based 3D viewer with clear developer instructions for local deployment using Node.js and Python backends. The README and environment setup files are sufficient for reproducing the frontend and backend locally.

siibra-api offers RESTful access to siibra-python functionality. While still labeled as experimental, it includes Docker-based deployment options and OpenAPI documentation for endpoint testing.

While I did not independently install and run the full stack, the documentation and modular design suggest that the results described in the manuscript are reproducible with reasonable effort.

We thank the reviewer once again for this positive feedback and the highly valuable suggestions for improving the manuscript.

Point-to-point responses to final reviewer comments

Our responses are formatted in green.

Reviewer #1

I accept the authors' responses and recommend the manuscript for publication.

We thank the reviewer for this positive recommendation.

I would nevertheless like to make one general remark concerning cytoarchitectonic reference atlases. Even when described as "observer-independent" and published in peer-reviewed high-profile journals, such atlases are rarely subjected to broad collegial expert evaluation within the specialized cellular neuroanatomy community. In practice, their widespread citation does not necessarily reflect consensus endorsement, but rather their availability, integration into major infrastructures, and practical convenience for the neuroimaging community, which lacks however expertise to assess cyto-architecture and accepts atlases for granted.

In this context, the Jülich atlas has achieved high visibility and adoption, particularly through its integration within EBRAINS and related platforms. This visibility, however, should not be conflated with universal acceptance as a consensus cytoarchitectonic standard. The cellular neuroanatomy community remains fragmented, and no single parcellation—including Jülich—has achieved full disciplinary consensus.

This does not invalidate the present manuscrip and SUBRA is truly an achievement a great service to the community, but it is an important contextual point when discussing the status and authority of reference atlases within multimodal neuroinformatics frameworks.

We appreciate this open and critical assessment. We have carefully reviewed the text to ensure the manuscript does not include generic statements regarding the authority of the Julich-Brain atlas, and would like to emphasize that we intentionally did not claim it represents a community standard. However, it is the standard cytoarchitectonic reference atlas used in EBRAINS by design decisions of the consortium. We are convinced that the coming years will help to overcome the fragmentation mentioned by the reviewer, and hope that the siibra tool suite can contribute to reaching this goal.

Reviewer #2

The authors have substantially revised the manuscript in response to the first-round comments, and the improvements are clear. The revised version provides a more rigorous and transparent presentation of siibra as a multilevel neuroinformatics platform for integrating heterogeneous human brain datasets into a harmonized atlas framework. The additions strengthen the manuscript's clarity, methodological grounding, and practical utility.

We thank the reviewer for this positive assessment.

Summary of Key Results: The manuscript now more effectively demonstrates how siibra unifies multimodal data, from MRI-scale templates to microscopic histology, within a consistent anatomical and coordinate framework. The expanded descriptions of reference spaces, parcellations, and data-access mechanisms improve the reader's understanding of how the platform supports interactive exploration, reproducible workflows, and multiscale analyses. Of note, addition of the hierarchical region tree to the siibra-python documentation is helpful.

Originality and Significance: The work remains technically original and fills a critical infrastructure gap in multilevel human brain data integration. The authors' emphasis on interoperability, extensibility, and integration of high-resolution modalities (e.g., BigBrain) continues to distinguish siibra from existing atlas-based frameworks. The revisions further highlight the platform's alignment with FAIR principles and its value to the broader neuroinformatics community.

Data and Methodology: The authors have addressed the earlier request for clearer validation and methodological transparency. The newly added quantitative assessments of spatial transformations, expanded parcellation overview, and detailed explanation of template switching and uncharted regions significantly strengthen the methodological section. The inclusion of performance benchmarks and response-time analyses provides useful insight into scalability and practical constraints. It was also interesting to read how the response times compare across the globe! Overall, the methodological presentation is now more complete and robust.

Statistics and Uncertainties The manuscript now incorporates quantitative evaluations of coordinate-mapping precision and system performance, appropriately characterizing uncertainties inherent in nonlinear spatial transformations and cloud-based data retrieval. These additions address the earlier concern that operational robustness was described only qualitatively. The authors added the following case study: Anatomical evaluation of subcortical maps, which greatly enriched the manuscript.

Conclusions: The conclusions are now better supported by the expanded analyses and clarifications. The platform is shown to be robust across multiple use cases, and the authors clearly articulate both its strengths and its current limitations. The manuscript now provides a more convincing case for the reliability and extensibility of the siibra framework.

Suggested Improvements: No further suggestions.

References: The references remain appropriate and comprehensive, with proper credit given to foundational resources such as BigBrain, Julich-Brain, and EBRAINS.

Clarity and Context: The manuscript is clearly written, and the revisions have improved the lucidity of the abstract, introduction, and methods. The added explanations and updated figures enhance accessibility for new users and strengthen the overall presentation.

We thank the reviewer for the thorough and constructive evaluation, and are pleased that the revisions have satisfactorily addressed the previous concerns.
